# Qualitative interviews of patients with COPD and muscle weakness enrolled in a clinical trial evaluating a new anabolic treatment: patient perspectives of disease experience, trial participation and outcome assessments

**DOI:** 10.1186/s41687-024-00712-0

**Published:** 2024-04-20

**Authors:** Maggie Tabberer, Nicola Williamson, Sophi Tatlock, Adam Gater, Rebecca Grimes, Chika Akinseye, David Neil, Aoife Mahon-Smith, Linda Nelsen

**Affiliations:** 1 GSK, Brentford, Middlesex UK; 2 PCO, Adelphi Values Ltd, Bollington, Cheshire, UK; 3 GSK R&D, Stevenage, Hertfordshire UK; 4 GSK R&D, 1250 S Collegeville Road, 19426 Collegeville, PA USA

**Keywords:** Chronic obstructive pulmonary disease, Muscle weakness, Qualitative interview, Anabolic treatment, Trial participation, Patient experience, Performance outcome assessment, Exit interviews

## Abstract

**Background:**

Chronic obstructive pulmonary disease (COPD) and muscle weakness can cause impaired physical function, significantly impacting patients’ health-related quality of life (HRQoL). Loss of muscle strength is usually assessed through clinical and performance outcome (PerfO) assessments, which consists of tasks performed in a standardized manner, providing evidence of a patient’s functional ability. However, evidence documenting the patient experience of COPD and muscle weakness is limited.

**Methods:**

This two-stage qualitative study used semi-structured interviews in patients aged 45–80 years with COPD (post-bronchodilator forced expiratory volume in 1s [FEV_1_]/forced vital capacity ratio < 0.70, and FEV_1_% predicted of 30–80%) and muscle weakness. In Stage 1, 30-minute concept elicitation interviews were conducted with participants recruited across three US sites to explore impacts on physical functioning and activities of daily living. In Stage 2, interviews were performed with participants exiting a Phase IIa trial investigating the efficacy of a selective androgen receptor modulator (GSK2881078) on leg strength, whereby PerfOs were used to evaluate strength and physical functioning endpoints. These participants completed either 60-minute in-depth (*n* = 32) or 15-minute confirmatory (*n* = 35) interviews exploring trial experience, completion of outcome measures, disease experience and treatment satisfaction.

**Results:**

In Stage 1 (*n* = 20), most participants described their muscles as weak (83.3%). Difficulties with walking (100%) and lifting heavy objects (90%) were reported. In Stage 2, 60-minute interviews, all participants (*n* = 32) reported a positive trial experience. Most participants reported that the home exercise program was easy to fit into daily life (77.8%), the PROactive daily diary was easy to complete (100%) and wearable sensors were easy to use (65.6%). However, technical issues were reported (71%), and few participants (19.4%) found physical assessments easy to complete. Improvements in muscle strength and functional limitations were reported by most participants. The shorter 15-minute confirmatory interviews (*n* = 35) supported the in-depth interview results.

**Conclusion:**

The qualitative interviews generated in-depth evidence of key concepts relevant to patients with COPD and muscle weakness and support the assessments of patient strength and physical function as outcome measures in this population in future studies.

**Trial number:**

GSK Stage 1: 206869; Stage 2: 200182, NCT03359473; Registered December 2, 2017, https://clinicaltrials.gov/ct2/show/NCT03359473.

**Supplementary Information:**

The online version contains supplementary material available at 10.1186/s41687-024-00712-0.

## Background

Muscle weakness, or cachexia, due to loss of muscle mass and strength, is a serious consequence of many chronic diseases, including chronic obstructive pulmonary disease (COPD) [1], a progressive respiratory disease characterized by airflow limitation and persistent respiratory symptoms [2].

In patients with COPD, functional inspiratory muscle weakness has been associated with worsening of dyspnea, or breathlessness [3, 4], which can lead to exercise limitation and thus further weakening of the muscles [5]. As such, muscle weakness can contribute to significant morbidity and early mortality [1, 6, 7] and lead to impaired physical function and mobility, significantly impacting patients’ health-related quality of life (HRQoL) [4, 8].

Currently, there are no approved treatments to improve muscle weakness with chronic conditions such as COPD, representing a major unmet clinical need. Although strength/resistance training improves physical function [9, 10], adjunctive therapies are required to promote rehabilitation, reduce the rate of physical decline and improve overall functioning [11–13]. Selective androgen receptor modulators (SARMs) bind to the androgen receptor and display antagonism in androgenic tissues [14–17], providing some anabolic effects that may help treat muscle weakness [18], without the disadvantages of testosterone, such as an increase in cardiovascular adverse events [19]. In clinical trials, SARMs have been shown to selectively increase lean body mass and are generally well tolerated [20–22].

In clinical trials, strength loss associated with loss of muscle mass and the associated impacts on physical functioning and HRQoL are best assessed from the patient perspective using clinical outcome assessments (COAs) [23]. COAs include patient-reported outcome (PRO) measures and performance outcome (PerfO) assessments, the latter being standardized tasks performed by a patient according to instructions administered by a trained individual such as a healthcare professional, or completed by the patient independently [23]. PerfOs can identify objective changes in muscle strength and functional performance to evaluate treatment benefits. Historically, PerfOs that have been used to assess functional limitations in COPD and muscle weakness include the 6-minute walk test (6MWT) [24], endurance and incremental shuttle walk tests (ESWT, ISWT) [25, 26], quadricep and handgrip strength tests [27, 28] and dynamometry. PerfOs can have substantial value as measures when the concepts of interest being evaluated best suit task performance and using a combination of COAs may provide a more complete picture of the patient’s experience of a disease and totality of evidence [29].

A lack of qualitative research documenting the patient experience of COPD and muscle weakness and its impact on physical functioning, activities of daily living (ADL) and HRQoL is evident. However, findings from quantitative studies suggest exercise endurance and tolerance [24–26], and strength and physical fitness [27, 28, 30–33], are relevant and important concepts.

The objective of this study was to conduct qualitative research with patients to identify impacts of muscle weakness associated with COPD on physical functioning, ADL, and HRQoL from the patient perspective. In particular, the current study addresses this lack of qualitative evidence using a two-staged approach. First, qualitative concept elicitation (CE) interviews were conducted exploring patients’ conceptualization of muscle weakness and its impact on physical functioning, ADL and HRQoL. Second, qualitative in-trial interviews were conducted with patients participating in a Phase IIa clinical trial (GSK: 200182; NCT03359473) [34] to explore disease experience including symptoms and HRQoL impacts, and any changes observed, treatment satisfaction, and feedback on outcome assessments.

## Methods

### Stage 1: CE interviews

#### Study design

A cross-sectional, qualitative study involving semi-structured CE telephone interviews was conducted in three sites across the USA between March and December 2018 with English-speaking patients with COPD and muscle weakness. Sampling quotas were employed to ensure good representation of patients by age, sex, muscle weakness severity, exacerbation history since COPD diagnosis, pulmonary rehabilitation use and hospitalizations within the past year. Ethical approval was provided by a centralized Independent Review Board in the USA, prior to conduct of any study-related activities (reference number: ADE1-18-360). All participants provided written informed consent.

#### Participant inclusion and exclusion criteria

All participants were required to be 45–80 years of age with clinician-confirmed COPD diagnosis (post-bronchodilator forced expiratory volume in 1 s [FEV_1_]/forced vital capacity ratio < 0.70, and FEV_1_% predicted of 30–80%) and clinician-confirmed diagnosis of muscle weakness associated with COPD. Further details of the eligibility criteria, and details of the recruitment and interview procedures are included in the Supplementary Materials.

### Stage 2: exit interviews

#### Study design

Qualitative exit interviews were conducted upon completion of the efficacy portion of a Phase IIa randomized, placebo-controlled, double-blind, parallel group study (GSK: 200182; NCT03359473), which evaluated the effects of the SARM GSK2881078 on leg strength in patients with COPD and muscle weakness in the USA, UK and Germany [34].

The 200182 trial design has been reported previously [34]. Postmenopausal women and men with COPD 50–75 years of age were enrolled if they met eligibility criteria, described in detail elsewhere. Participants were randomized 1:1 to either GSK2881078 (women: 1 mg; men: 2 mg) or matching placebo, orally once daily for 13 weeks, alongside a standardized home exercise program, Respercise, developed by GSK, based on the SPACE rehabilitation program from the University of Leicester, UK [35], and delivered via a smartphone app [34].

#### Trial outcome assessments

Details of the 200182 trial assessments can be found in the Supplementary Materials, Supplementary Fig. 1 and Supplementary Table 1, and have been described previously [34]. Briefly, for the 13-week home exercise program participants used the Respercise app. An additional daily physical activity goal was set according to participants’ baseline and daily performance, based on input of daily step counts via a wrist-worn activity tracker (Vivofit).

Participants’ physical activity was rated and measured using the daily PROactive Physical Activity in COPD instrument (D-PPAC) [31, 32], a hybrid tool comprising a daily questionnaire and measured outputs from a triaxial physical activity monitor (GT9X) with the device worn on the waist for 7 days at four time points (screening, baseline [Day − 9], Day 56 and Day 80).

Participants also performed physical assessments during site visits, including the ISWT [26] and the ESWT [25, 36, 37]. In addition, spirometry was performed according to ATS/ERS guidelines [38]. Inspiratory muscle strength was also assessed by measuring the maximal sniff nasal inspiratory pressure (SnIP) [39]. Lean body mass was assessed using a dual-energy x-ray absorptiometry (DXA) scan.

#### Exit interview procedures

**Fig. 1:**
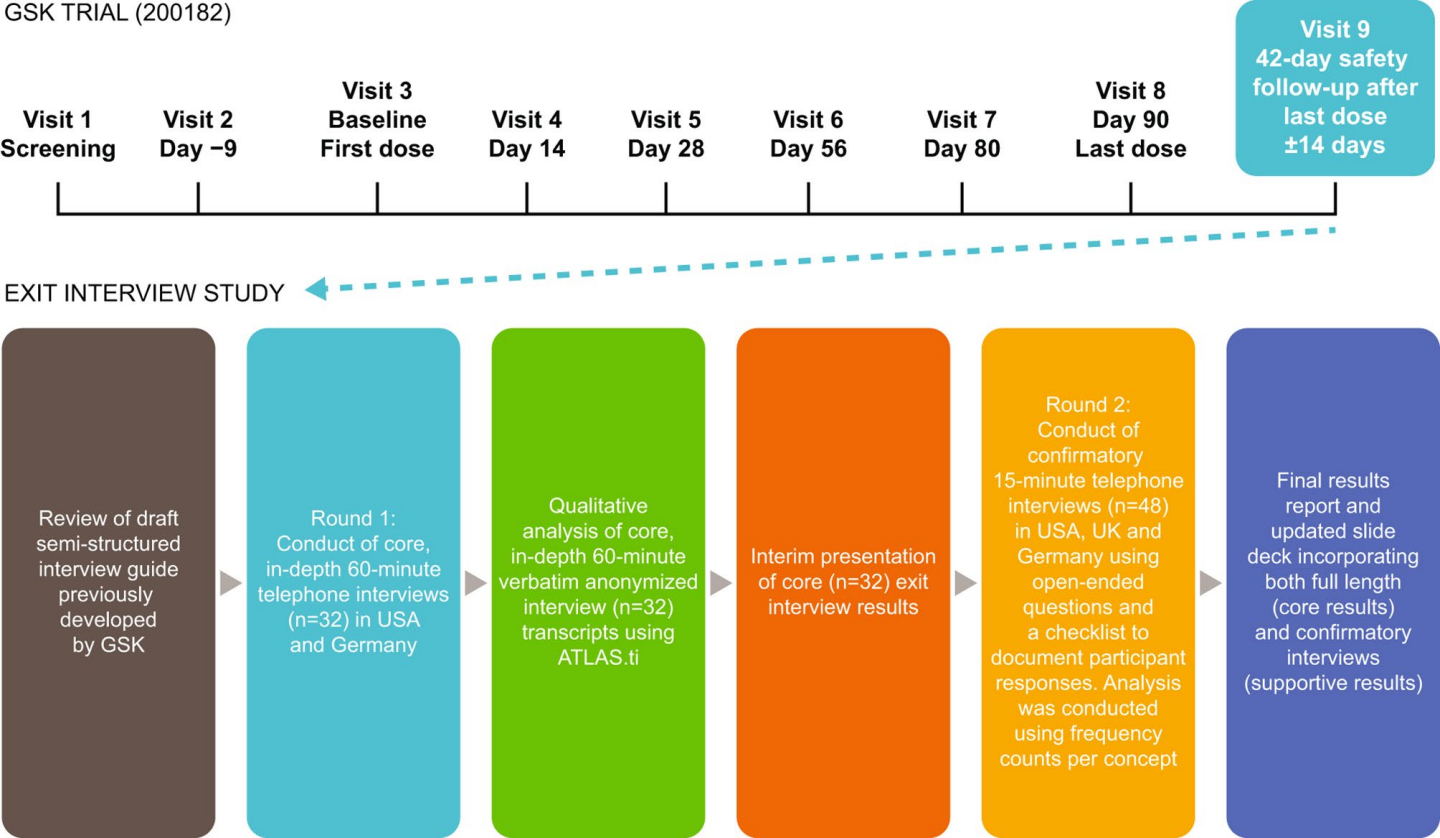
Exit interview study methodology

All patients who completed the final study visit of the efficacy phase of the 200182 trial, were eligible for participation in an exit interview (at Visit 9, 42 days post last dose; Fig. 1) [34] between August 2018 and November 2019. A single informed consent form was used to consent patients to participate in both the clinical trial and exit interviews. Verbal consent was additionally obtained prior to the interview.

An overview of the exit interview study methodology is detailed in Fig. 1. In accordance with guidance for establishing conceptual saturation in qualitative samples, which suggests that 99% of concepts are likely to be elicited after 25 interviews [40] and consideration of the heterogeneity of the participant population, full-length, in-depth 60-minute interviews were conducted with the first 32 participants who completed the efficacy phase of the study (in the USA and Germany). All remaining participants who subsequently completed the study (35 participants in the USA, UK and Germany) participated in a shorter, confirmatory, 15-minute interview in which the relevance of concepts identified within the in-depth interviews was explored.

A semi-structured interview guide was developed to guide discussion and ensure topics of interest were covered during the 60-minute interviews. The interview guide contained open-ended CE questions designed to elicit feedback on the following key areas: clinical trial experience including the enrollment process, trial procedures and information received; trial outcome assessments completed (i.e., home exercise program, wearable sensors [Vivofit and GT9X], PROactive daily diary [the D-PPAC] [28, 29] and physical assessments [ISWT [24], ESWT [23, 34, 35], SnIP [35] and DXA]); disease experience, including symptom presentation, functional ability and impacts experienced by participants before the trial, whether these concepts changed during the trial and how meaningful these changes were; and treatment satisfaction, including changes in disease characteristics, and satisfaction with medication received.

All interviews were blinded and conducted by experienced qualitative interviewers in the native language of the respective country. All interviews were audio recorded, but only 60-minute interview recordings were transcribed verbatim, with findings from 15-minute interviews documented via an interviewer-completed checklist. Interviews conducted in German were transcribed and translated into US English by an accredited translation agency for analysis.

### Stage 1 and 2 qualitative analysis

Participant demographic and clinical characteristics were summarized using descriptive statistics. Qualitative analyses were conducted using ATLAS.ti version 7 (ATLAS.ti Scientific Software Development GmbH, Berlin, Germany).

Qualitative analysis of all interview transcripts was conducted using thematic analysis methods [41]. All interviews were analyzed iteratively as they were conducted. The coding process was blinded and the first two coded interview transcripts for each stage were reviewed by the same research team to confirm the suitability of the coding structure and ensure consistent application and grouping of codes across transcripts. Following the review and coding of all qualitative data, all coded data, themes and supporting quotes were extracted from ATLAS.ti into an Excel spreadsheet to support further analysis and filtering of data for reporting purposes.

In Stage 2, descriptive statistics were performed on data collected from 15-minute interviews, to examine whether findings were consistent with those from 60-minute interviews. No formal qualitative analysis was conducted for 15-minute interviews as the aim for these was to confirm concepts reported in the initial 60-minute in-depth interviews. Frequency counts were therefore reported.

## Results

### Stage 1: CE interviews

#### Demographic and clinical characteristics

Twenty participants participated in the CE interviews. The sample included more females (*n* = 12; 60.0%), with a mean (standard deviation [SD]) age of 64.2 (9.5) years; range 48–79 years. Mean (SD) months since COPD and muscle weakness diagnosis was 45.1 (27.6) and 22.9 (15.4) months, respectively (Table 1).

**Table 1:** Baseline demographic and clinical characteristics in the concept elicitation interviews of Stage 1

	Total (*N* = 20)
**Age (years), mean (SD)**	64.2 (9.5)
**Female, n (%)**	12 (60.0)
**Race, n (%)**	
White/Caucasian Black African or Caribbean	10 (50.0) 10 (50.0)
**Clinical characteristics**	
**Time since COPD diagnosis (months), mean (SD)**	45.1 (27.6)
**Time since muscle weakness diagnosis (months), mean (SD)**	22.9 (15.4)
**Baseline predicted FEV**_**1**_ **(%), mean (SD)**	50.1 (11.2)
**Predicted FEV**_**1**_, **n (%)**	
20–29% 30–39% 40–49% 50–59% 60–69% 70–80%	1 (5) 2 (10) 4 (20) 10 (50) 2 (10) 1 (5)
**BMI (kg/m** ^**2**^ **), mean (SD)**	26.6 (3.6)
**COPD exacerbations since diagnosis, n (%)**	
0 1 2 3	6 (30.0) 9 (45.0) 3 (15.0) 2 (10.0)
**Patients hospitalized in the past 12 months, n (%)**	
0 1 2	15 (75.0) 3 (15.0) 2 (10.0)
**CAT score, mean (SD)**	20.3 (6.9)
**Current treatments for COPD, n (%)**	
ICS LABA LAMA	19 (95.0) 20 (100.0) 11 (55.0)
**Severity of muscle weakness condition, n (%)**	
Mild Moderate Severe	8 (40.0) 9 (45.0) 3 (15.0)

BMI, body mass index; CAT, COPD Assessment Test; COPD, chronic obstructive pulmonary disease; FEV_1_, forced expiratory volume in 1 s; ICS, inhaled corticosteroids; LABA, long-acting β_2_-agonist; LAMA, long-acting muscarinic antagonist; SD, standard deviation

Mean FEV_1_% predicted was 50.1% and most participants had experienced one (*n* = 9; 45.0%) or no (*n* = 6; 30.0%) exacerbation since their COPD diagnosis. Most participants had clinician-reported mild (*n* = 8; 40.0%) or moderate (*n* = 9; 45.0%) muscle weakness associated with COPD (Table 1).

#### CE interviews

Denominator values differed between topics, depending on what patients discussed in the interviews. Most participants described their muscles as ‘weak’ (*n* = 15/18; 83.3%). Additionally, most participants reported changes to their muscle strength over time following diagnosis of COPD (*n* = 19/20; 95.0%), with 8 (42.1%) participants describing their muscles as becoming weaker (“*I end up feeling more weaker and I get the cramps*”), 6 (31.6%) as becoming worse, 2 (10.5%) as sore and 3 (15.8%) as no change. Only 1 (5.3%) participant reported their muscles becoming stronger (“*They’re probably getting a little stronger now…I’ve been moving a lot more… [before their diagnosis] [I] had many problems getting winded once I were to pick something up. But now I seem to be getting a lot better*”). One participant used two descriptors so the total number of responses exceeded 19.

Participants reported difficulties performing physical movements due to COPD and muscle weakness, with walking difficulties (*n* = 20/20; 100%), lifting or carrying heavy objects (*n* = 18/20; 90.0%; *“I am unable to lift as much as I could before”*), and climbing stairs (*n* = 17/20; 85.0%) most commonly mentioned. A similar proportion of participants attributed their walking difficulties to either respiratory symptoms of COPD alone (*n* = 5/12; 42.0%) or both respiratory symptoms and muscle weakness associated with COPD (*n* = 5/12; 42.0%).

The most common ADL impacted were housework (*n* = 17/20; 85.0%), exercise (*“Unfortunately I cannot run. Running is out of the question. I’m getting to the point where I can’t walk a long distance and that’s what scares me”*) and personal care (both *n* = 14/20; 70.0%). Many participants described a change over time in their motivation to complete ADL and having to push themselves more (*n* = 13/17; 77.0%; “*I would really have to push myself to do most any activity myself”*). Some participants also noted difficulties with leaving the house (*n* = 6/16; 37.5%).

Impacts on HRQoL reported by participants (*n* = 20/20; 100%) affected social life (*n* = 16/20; 80.0%), including meeting with friends (*n* = 6/16; 37.5%) (“*I used to go walking a lot with my girlfriends and I can’t do that any longer”*), going out with family (*n* = 2/16; 12.5%) and going dancing (*n* = 2/16; 12.5%). HRQoL impacts of muscle weakness were also reported to negatively affect emotional functioning (*n* = 11/20; 55.0%), relationships (*n* = 10/20; 50%) and work (*n* = 8/20; 40.0%). For example, participants reported not being able to do things with friends and family (*n* = 4), relying heavily on partners (*n* = 2; “*It’s just that, you know, it bothers me that my wife has to do everything now*.”), avoiding making relationships/pushing people away (*n* = 2), seeing less of their family (*n* = 1) and their friends not understanding their condition (*n* = 1). Work was also impacted by participants not being able to stand for a long time (*n* = 2), experiencing muscle cramps from sitting for too long (*n* = 2; “*I can’t sit a long period of time because that bothers me. That makes me stiff and numb and the muscles act*.”), struggling to lift objects (*n* = 1), being unable to walk far (*n* = 1) and not being able to perform well (*n* = 1).

### Stage 2: exit interviews

#### Demographic and clinical characteristics

Sample attrition for the 200182 study has been reported previously [34]. In total, 97 participants were randomized into the 200182 study and overall, 76 completed all required study visits; however, 9 participants refused to participate in an exit interview. The remaining 67 participants took part in an exit interview. The target sample of 32 in-depth 60-minute interviews was achieved, while 35 further participants took part in the 15-minute confirmatory interviews. Participant demographics and clinical characteristics were similar between the overall trial population and those who participated in the 60-minute exit interviews (*n* = 32; Table 2; Supplementary Materials).

**Table 2:** Baseline demographics and clinical characteristics of the 200182 trial population and the exit interview population

Demographics	Total clinical trial (*N* = 96)	Total exit interview (*N* = 67)	60-minute interview (*n* = 32)	15-minute interview (*n* = 35)
**Age (years), mean (SD)**	65.1 (7.1)	65.1 (7.2)	64.6 (8.2)	65.5 (6.5)
**Female, n (%)**	47 (49.0)	36 (53.7)	14 (43.8)	22 (62.9)
**Race n (%)**				
White/Caucasian/European African American/African	91 (94.8) 7 (5.2)	64 (95.5) 3 (4.5)	29 (90.6) 3 (9.4)	35 (100.0)
**Country, n (%)**				
USA Germany UK	51 (53.1) 33 (12.5) 12 (12.5)	39 (58.2) 19 (28.4) 9 (13.4)	24 (75.0) 7 (18.8) 2 (6.3)	15 (42.9) 13 (37.1) 7 (20.0)
**Clinical characteristics**				
**Baseline predicted FEV**_**1**_ **(%), mean (SD)**	48.8 (10.8)	50.3 (10.7)	52.3 (11.3)	48.4 (9.8)
**BMI (kg/m** ^**2**^ **), mean (SD)**	25.0 (4.2)	25.2 (4.2)	24.3 (3.9)	26.1 (3.9)
**CAT score, mean (SD)**	18.4 (6.5)	18.6 (6.6)	18.6 (6.4)	18.6 (6.9)
**Trial treatment arm, n (%)**				
Treatment Placebo	49 (51.0) 47 (49.0)	35 (52.2) 32 (47.8)	17 (53.1) 15 (46.9)	18 (51.4) 17 (48.6)

BMI, body mass index; CAT, COPD Assessment Test; COPD, chronic obstructive pulmonary disease; FEV_1_, forced expiratory volume in 1 s; ICS, inhaled corticosteroids; LABA, long-acting β_2_-agonist; LAMA, long-acting muscarinic antagonist; SD, standard deviation

### Exit interviews

#### 200182 trial experience

Most participants of the 60-minute (*n* = 32/32; 100%) or 15-minute interviews (*n* = 34/35; 97.1%) reported a positive trial experience, most commonly related to the level of information, support and communication provided by site staff (*“…the staff is, is really fabulous…they’re very informative, make sure that I totally understand everything*”). All participants in the 60-minute interviews (*n* = 32/32; 100%) reported that the enrollment process was clear and they understood what was involved in the trial prior to consent. Additionally, all participants asked (*n* = 25/25; 100%) reported positive perceptions of the trial duration and would participate in future trials. Findings were supported by the 15-minute interviews, with only 1 (1/35 [2.9%]) and 2 (2/21 [9.5%]) participants stating that the trial duration was too long, or they would not participate in future trials, respectively.

Fewer participants reported negative aspects of their trial experience (*n* = 16/67; 23.9%). In the 60-minute interviews, 2 participants reported disliking the DXA body scan procedures (*n* = 2/32; 6.3%), 1 participant disliked giving blood samples, and another felt that the SnIP instructions were unclear (*n* = 1/32; 3.1% each). Participants in the 15-minute interviews also reported negative aspects of the trial experience (*n* = 12/35; 34.3%), most commonly experiencing side effects and participating in the home exercise program (both *n* = 3/35; 8.6%).

#### 200182 trial outcome assessments

**Fig. 2:**
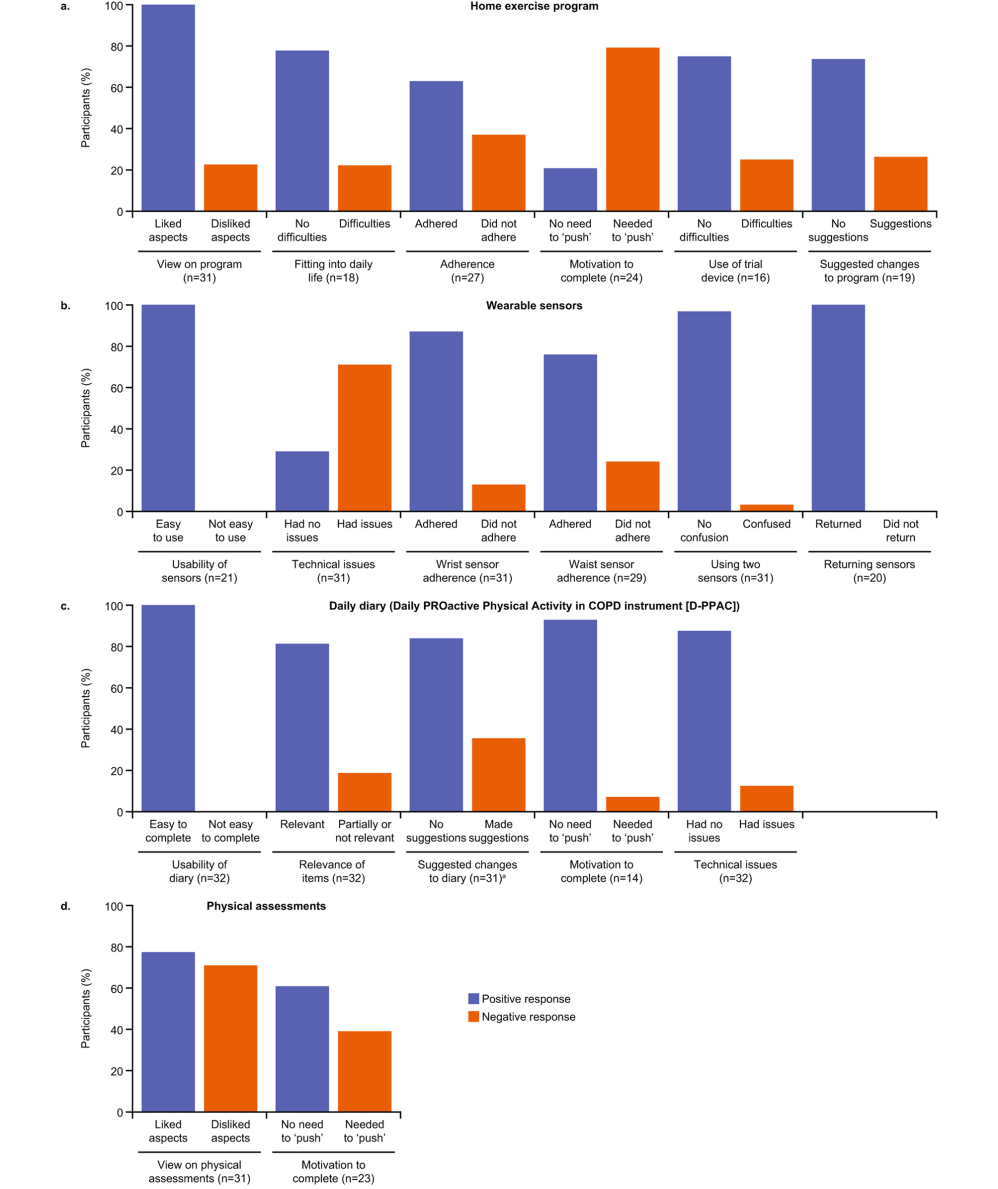
Feedback on 200182 trial outcome assessments during the 60-minute (*n* = 32) exit interviews. ^a^Participants provided more than one response so counts exceed totals. COPD, chronic obstructive pulmonary disease

Participants described their experiences of completing the trial outcome assessments, including the home exercise program, wearable sensors, PROactive daily diary and the physical assessments completed at site visits (Fig. 2 and Supplementary Table 3).

#### Home exercise program

All participants who discussed the home exercise program (*n* = 31/32; 96.9%) in 60-minute interviews provided positive feedback, with 6 participants reporting feeling stronger as a result. Most participants reported that home exercise was easy to fit into their daily life (*n* = 14/18; 77.8%; “…*they weren’t hard to do at all and it didn’t take but a few minutes at night before I go to bed”*) and self-reported adherence to the home exercise program was high (*n* = 17/27; 63.0%). However, 7 participants reported disliking certain aspects, with walking exercises the most commonly cited (*n* = 3/7; 42.8%; “*The exercises were simply tedious…I did the exercises, but they were boring”*). Additionally, most participants (*n* = 19/24; 79.2%) reported ‘pushing themselves’ to complete the program, particularly at the start of the trial (*n* = 8/24; 33.3%), although motivation improved with a regular routine.

In 15-minute interviews, most participants (*n* = 31/35; 88.6%) also reported that the home exercise program was easy to complete and fit into their daily life. Four participants reported that the program was difficult to complete, all of whom were between 70 and 76 years of age.

#### Wearable sensors

Participants in both interview groups reported mixed feedback on the wrist and waist wearable sensors. Most participants asked in 60- and 15-minute interviews described the sensors as easy to use (*n* = 21/32, 65.6%; *n* = 28/34, 82.4%, respectively), with no confusion using two different wearable sensors (*n* = 30/31, 96.8%; *n* = 32/34, 94.1%, respectively). In addition, self-reported adherence to wearing the sensors was reported to be good in 60-minute interviews with most participants (*n* = 27/31, 87.1%; *n* = 22/29, 75.9%) wearing the wrist and waist sensors, respectively, at all times during the required trial period (*“…I did not take it off except to take a shower”*).

However, a range of technical issues were reported during 60-minute interviews, the most common being that the waist tracker did not feel securely attached to the belt provided (*n* = 11/31; 35.5%), resulting in participants feeling concerned or worried that they would lose the sensor during the trial (*“There was no way to secure it without, uh, without putting it in my pocket”*). Similarly, in 15-minute interviews, 16/35 (45.7%) participants reported experiencing technical difficulties, including the waist sensor falling off (*n* = 5/35; 14.3%), and the wrist sensor not accurately recording steps (*n* = 3/35; 8.6%).

#### PROactive daily diary

All participants in the 60-minute interviews (*n* = 32/32; 100%) and most participants in the 15-minute interviews (*n* = 33/35; 94.3%) reported that the PROactive daily diary was easy to complete. Most participants (*n* = 26/32; 81.3%) in the 60-minute interviews reported that PROactive diary items were relevant to their disease experience (*“I think they were the relevant questions which should be asked to gain a picture of my condition”*), whereas in the 15-minute interviews more participants (*n* = 11/34; 32.4%) reported that some PROactive daily diary items were not relevant. Reasons for lack of relevance included some items not being suitable for participants who had none or mild physical limitations or that items were too general in scope.

#### Physical assessments

Participants in both interview groups provided mixed feedback about the physical assessments completed during trial visits. Fewer participants in the 60-minute interviews (*n* = 6/31; 19.4%) reported that the physical assessments were easy to complete compared with those in the 15-minute interviews (*n* = 26/33; 78.8%). Some tests were described as challenging and/or difficult to complete by participants (*n* = 16/24; 66.7%) in the 60-minute and 15-minute (*n* = 7/33; 21.2%) interviews. Assessments most commonly reported to be challenging in both the 60- and 15-minute interviews included the leg press (*n* = 14/24, 58.3%; *n* = 21/33, 63.6%, respectively) and walking test (*n* = 5/24, 20.8%; *n* = 6/33, 18.2%, respectively; “*Only thing I hated was the leg press… It was really hard to do”*).

#### Disease experience

**Fig. 3:**
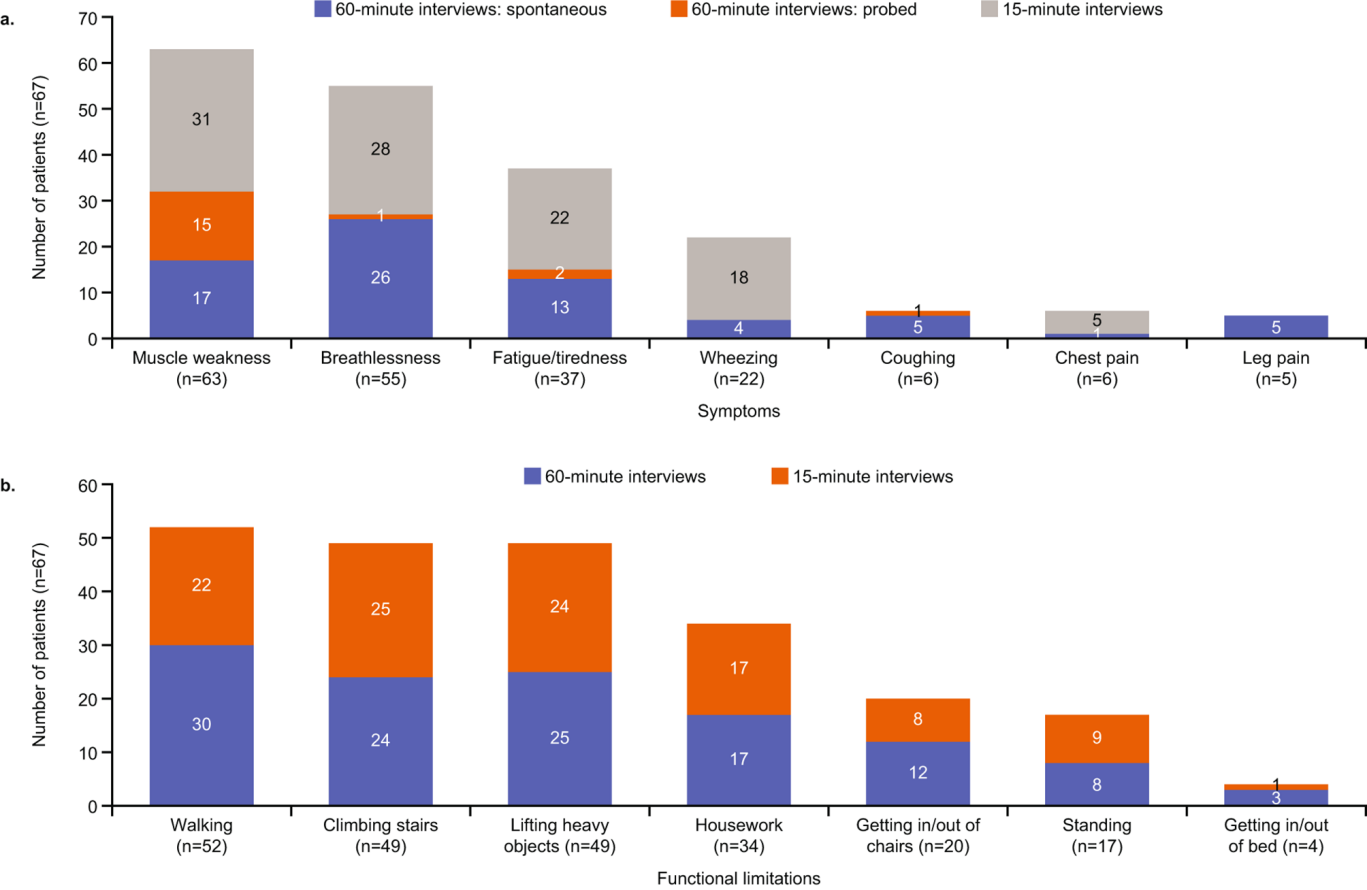
Overview of (A) symptoms and (B) functional limitations^a^. ^a^Reported by participants during the 60-minute (*n* = 32) and 15-minute exit interviews (*n* = 35) at the start of the trial. The remaining participants were either not questioned regarding the relevant symptom or limitation, or did not spontaneously mention it during the interview

Similar symptoms at the start of the trial were reported by participants across both interview groups, the most frequent being muscle weakness (*n* = 63/67; 94.0%), breathlessness (*n* = 55/67; 82.1%) and fatigue/tiredness (*n* = 37/67; 55.2%; Fig. 3A). A summary of the symptoms reported by participants in 60-minute interviews is provided in Supplementary Table 3.

**Fig. 4:**
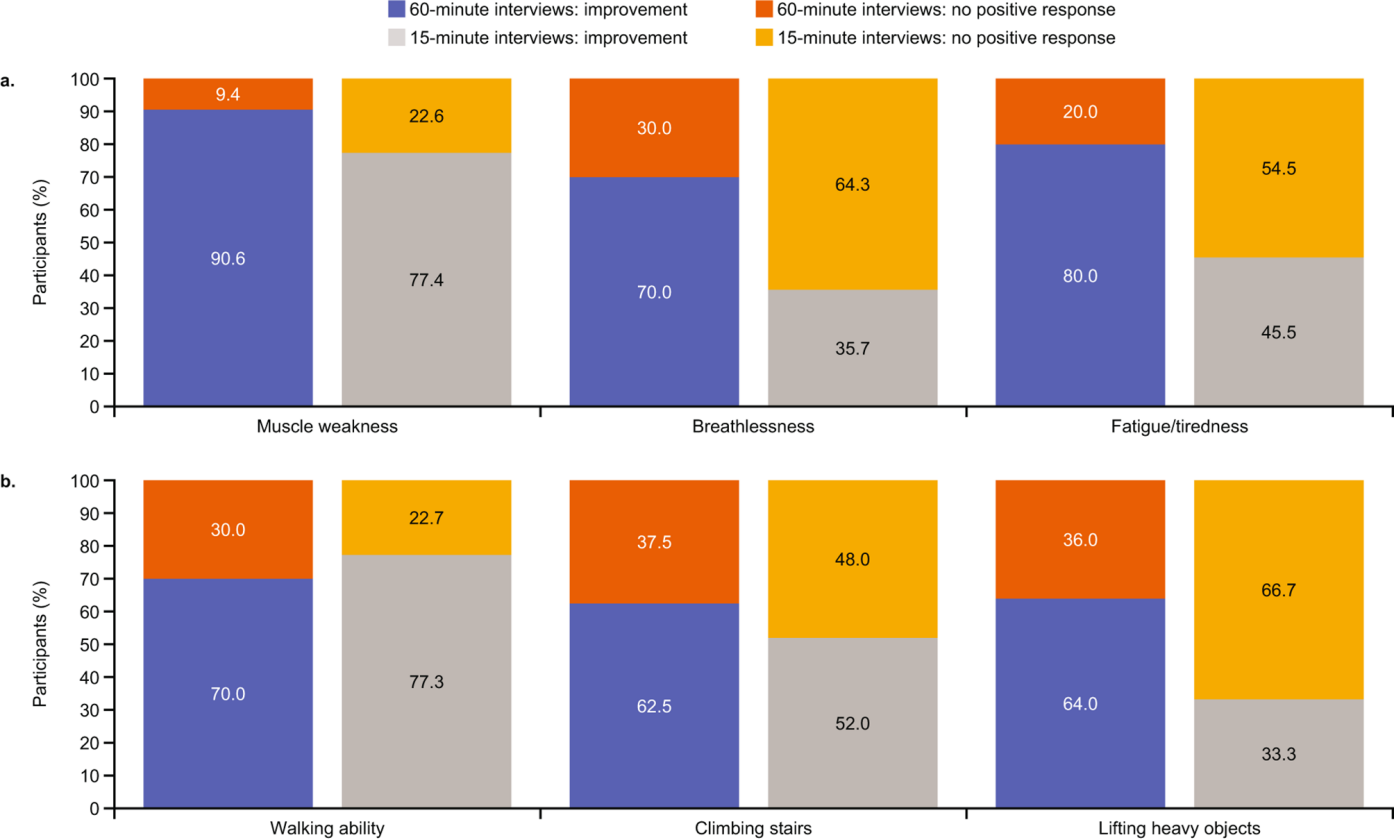
Overview of changes in (A) symptoms and (B) functional limitations during the trial^a^. ^a^Reported by participants during the 60-minute (*n* = 32) and 15-minute interviews (*n* = 35). No positive response included participants who either mentioned that there was no change or did not specifically comment on whether they had experienced a change

Most participants in both interview groups reported that during the trial they had experienced increased muscle strength (*n* = 43/51; 84.3%; *“I felt as though it allowed me to increase my arm strength and my leg strength”*), reduced breathlessness (*n* = 30/55; 54.5%) and less fatigue/tiredness (*n* = 22/37; 59.5%) than before the start of the trial (Fig. 4A).

Similar functional limitations were reported by participants in the 60- and 15-minute interviews. Participants most commonly reported limitations with walking (*n* = 52/67; 77.6%), climbing stairs (*n* = 49/67; 72.1%) and lifting heavy objects (*n* = 49/67; 73.1%; Fig. 3B), many of whom reported an improvement in these limitations at the end of the trial (walking: *n* = 38/52; 73.1%; climbing stairs: *n* = 28/49; 57.1%; lifting heavy objects: *n* = 24/49; 57.1%; Fig. 4B).

#### Trial satisfaction

Regardless of treatment arm, most participants reported feeling ‘very satisfied’ or ‘satisfied’ (*n* = 52/67; 77.6%) with changes to their symptoms during the trial. Participants most commonly reported feeling satisfied due to improved breathing (*n* = 7/67; 10.4%), increased overall strength (*n* = 5/67; 7.5%), more energy (*n* = 4/67; 6.0%) and increased leg strength (*n* = 3/67; 4.5%). Eleven (16.4%) participants reported feeling neither satisfied nor dissatisfied, three (4.5%) were dissatisfied and one (1.5%) very dissatisfied with the trial experience, but participants generally did not give a rationale for these responses.

Most participants across both treatment arms reported feeling ‘very satisfied’ or ‘satisfied’ (*n* = 48/66; 72.7%) with changes to their strength or physical ability during the trial. The most commonly reported reasons for this were increased general strength (*n* = 6/48; 12.5%), increased leg strength (*n* = 5/48; 10.4%), and increased arm strength (*n* = 5/48; 10.4%).

## Discussion

Muscle weakness is a common and debilitating phenomenon among patients with COPD. While research has sought to determine the molecular and biological pathways of skeletal muscle dysfunction in COPD [42], understanding of the direct patient experience and associated consequences (as captured via qualitative research methods) is limited. However, such information is critical to determine key concepts of interest and support measurement strategies for evaluating the efficacy of both pharmacological and non-pharmacological interventions (e.g., exercise training) in clinical studies.

Open-ended qualitative interviews conducted as part of Stage 1 CE interviews of this study provided detailed insights on the patient experience of living with COPD and muscle weakness. Consistent with published literature, exercise performance, strength, exercise tolerance and exercise capacity were identified as key concepts of COPD and muscle weakness impacting participants’ lives [43–46]. During the Stage 2 exit interviews, participants described qualitative improvements in their symptoms and functional limitations beyond those captured by COAs. Muscle weakness was the symptom most frequently reported at the start of the trial and was also reported to have improved most frequently by the end of the trial, supporting muscle strength as a meaningful aspect of patients’ daily lives. These Stage 1 and 2 findings provide initial evidence that the use of PerfOs such as the 6MWT [24], shuttle walk tests [25, 26], and hand grip and lower limb strength tests [27, 28], and assessments of physical activity such as the GT9X and PROactive daily diary may be appropriate for the assessment of strength and exercise performance, tolerance and capacity as part of a COA measurement strategy for clinical development programs in this population.

However, it is important that evidence is generated to establish the content validity of PerfOs. The US FDA Patient-Focused Drug development Guidance provide clear instructions on how content validity of PRO measures can be established, and broadly similar considerations can be applied to PerfOs [47]. More recently, an ISPOR Task Force on the selection, development and modification of PerfOs has provided detailed recommendations regarding evidence generation requirements for PerfOs to be fit-for-purpose in a specific context; debriefing of task performance of PerfO assessments can provide valuable insights regarding patient understanding of the relationship between the task and the meaningful aspect of health being measured [29]. Additionally, ecological validity is beneficial to assess the suitability of PerfO assessments for real-world functioning; however, the relevance of ecological validity is greater for those measures that have an indirect relationship to the aspect of health being investigated and should not be seen as a suitable replacement for qualitative data [29]. The Stage 2 exit interviews in this study provided an opportunity to explore the feasibility of and feedback on PerfOs completed by participants in the clinical trial. Overall, participants found each of the PerfOs easy to operate. Feedback on the home exercise program and PROactive daily diary were mostly positive, indicating that these trial outcome measures are feasible for assessing concepts identified as relevant to muscle weakness in Stage 1. While feedback was more mixed on the physical assessments, as participants reported difficulties completing the leg press and walking test, this also supports the relevance of these outcome measures for assessing concepts impacted by muscle weakness. Similarly, mixed feedback was also provided on the wearable sensors due to technical issues, highlighting the importance of using sensor technology that is fit-for-purpose to support trial endpoints. The use of wearables in clinical trials is rapidly increasing as they can offer huge value to outcomes by providing objective real-time data and allowing for smaller samples, shorter trials and improved clinical data. However, it is imperative that wearables demonstrate key components of verification, analytical validation and clinical validation; each component should demonstrate accuracy, reliability, precision and consistency over time and across different environmental conditions [48]. Wearable sensors also need to be easy and appropriate for participants to wear and reliably collect sensor data [48]; findings from these interviews highlight improvements that can be made to the wearable sensors ahead of use in future studies to support their clinical validation.

The Stage 2 exit interviews also provided the first opportunity to explore participants’ disease experience of a new treatment beyond COAs and biomarker assessments. The conduct of exit interviews within the context of a clinical trial is still a relatively new and evolving methodology but is growing in popularity as its potential value is being realized by key stakeholders, including regulators [47]. The methodology is increasingly being used as an additional way of capturing participant experiences and obtaining in-depth feedback on disease symptom changes, HRQoL impacts, evaluation of treatment benefit–risk and perspectives on clinical trial procedures and participation [49–55]. Such findings can inform trial designs, the refinement and/or interpretation of COAs for use in future trials and provide supplementary evidence to regulatory agencies to demonstrate treatment benefit [52, 55].

In addition to improvements in muscle weakness, participants in the treatment arm also described improvements in breathlessness and fatigue/tiredness during the trial; however, these improvements were reported in a slightly greater proportion of participants in the placebo arm (data not shown). It was not possible in this trial to ascertain whether there was a true treatment difference in changes in the symptom experience reported across both treatment arms. This could be because all trial participants completed the home exercise program as standard of care during the trial. However, these improvements in breathlessness and fatigue highlight the importance of strengthening muscles in patients with COPD, either through pharmacological or non-pharmacological methods, in order to improve symptoms that have a detrimental impact on patients’ quality of life.

Most participants across both the treatment and placebo arms reported being ‘very satisfied’ or ‘satisfied’ with the changes they experienced in symptoms and functional abilities, supporting the participant perception of the treatment efficacy of GSK2881078 in terms of improvements in symptoms and functional limitations in conjunction with standard of care exercise and providing supplementary evidence regarding treatment benefit. Overall, participant experiences of the 200182 trial were largely positive, indicating the design to be feasible, not overly burdensome to participants and of sufficient duration to capture changes and variability in participants’ experiences.

The large number of exit interviews conducted in Stage 2 (*n* = 67) is considered a strength of the study. The 60-minute interviews provided a means of gaining in-depth insights from patients, while the novel use of in-depth and confirmatory shorter qualitative interviews afforded the opportunity to explore participant experiences of the whole clinical trial sample in a feasible and pragmatic manner. The comparable findings across both interview groups indicate that conceptual saturation was achieved, supporting the adequacy of the sample size. Additionally, the demographic and clinical characteristics of the 60-minute exit interview sample were broadly similar to the total clinical trial sample [34], suggesting that results of these interviews are likely representative of the experience of the total trial sample, excluding early withdrawers. However, it is important to acknowledge study limitations in the interpretation of the results. The study only enrolled participants with COPD and muscle weakness and as such, may not be generalizable to participants with other chronic diseases with muscle weakness. While the findings provide some preliminary evidence for the content validity of the outcome measures used during the Phase IIa study, as participants were not formally debriefed on these measures further testing is required to ensure that all items are relevant for the target population. Additionally, the length of the 15-minute interviews meant that it was only possible to explore topics with relatively limited depth, meaning that some concepts were explored more fully than others. Additional insights may have been elicited if the 15-minute interviews were longer in duration, and if participants who withdrew from the study prior to completion were also included to investigate whether or not withdrawal was related to any of the study procedures. However, for this study it was not considered feasible to conduct in-depth interviews with the whole clinical trial sample so in-depth interviews supported by shorter confirmatory interviews were considered a realistic solution. Furthermore, it is possible that some participants in the US may have been native Spanish speakers whose English-language skills may have directly or indirectly resulted in them being excluded from participating in this study.

## Conclusion

The findings from the CE interviews identified key concepts relevant to patients experiencing muscle weakness associated with COPD. The exit interviews also provided support for the 200182 trial design, the relevance of the assessments to the patient experience, and provide preliminary content validity evidence for the PerfOs implemented. While further research is required to fully confirm the validity of the assessment measures for this patient population, the evidence generated through these interviews supports the assessment of muscle weakness through PerfOs as being important to the patient experience.

### Electronic supplementary material

Below is the link to the electronic supplementary material.

Supplementary Material 1

Supplementary Material 2

## Data Availability

**Stage 2**: Upon publication, anonymized individual participant data and study documents can be requested for further research from https://www.gsk-studyregister.com/en/ The study protocol is available on https://www.gsk-studyregister.com/en/trial-details/?id=200182.

## Abbreviations

1-RM: One repetition maximum
6MWT: 6-minute walk test
ADL: Activities of daily living
BMI: Body mass index
CAT: COPD Assessment Test
CE: Concept elicitation
COAs: Clinical outcome assessments
COPD: Chronic obstructive pulmonary disease
D-PPAC: Daily PROactive Physical Activity in COPD
DXA: Dual-energy x-ray absorptiometry
ESWT: Endurance shuttle walking test
FEV_1_: Forced expiratory volume in 1 s
FVC: Forced vital capacity
HRQoL: Health-related quality of life
ICS: Inhaled corticosteroids
IEC: Independent Ethics Committees
IRB: Institutional Review Boards
ISWT: Incremental shuttle walk test
LABA: Long-acting β_2_-agonist
LAMA: Long-acting muscarinic antagonist
PerfO: Performance outcome
PGIC: Patient Global Impression of Change
PGRS: Patient Global Rating of Severity
PRO: Patient-reported outcome
SARM: Selective androgen receptor modulator
SD: Standard deviation
SGRQ: St George’s Respiratory Questionnaire
SnIP: Sniff nasal inspiratory pressure
SPPB: Short Physical Performance Battery
