# Supplementary figures and images for: Qualitative interviews of patients with COPD and muscle weakness enrolled in a clinical trial evaluating a new anabolic treatment: patient perspectives of disease experience, trial participation and outcome assessments
Source: J Patient Rep Outcomes. 2024 Apr 20;8:45. doi: 10.1186/s41687-024-00712-0 (PMC11031513; doi:10.1186/s41687-024-00712-0)

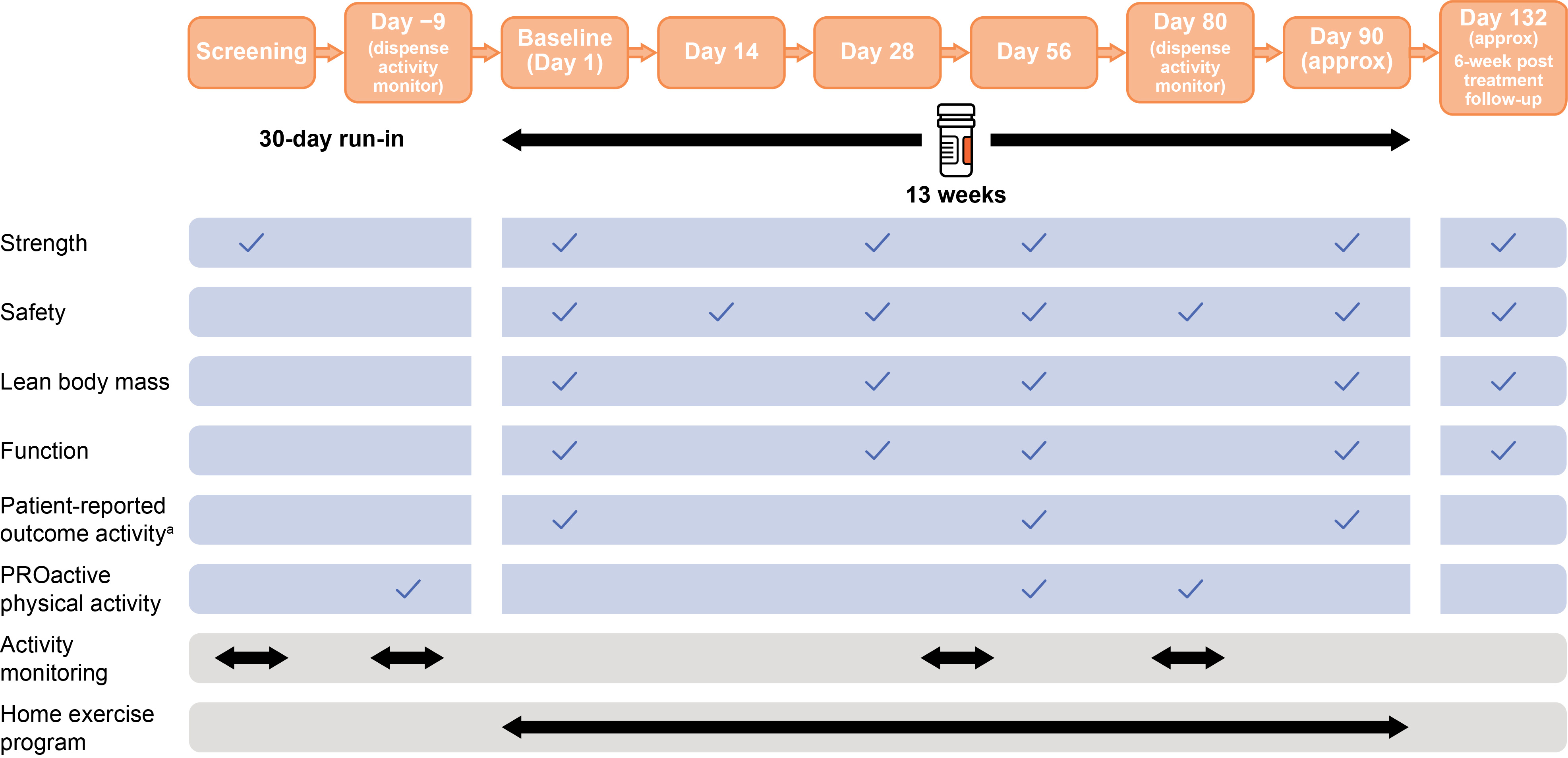

Supplement: Supplementary file 1 — Supplementary Material 1 [file 41687_2024_712_MOESM1_ESM.jpg]
