# Supplementary material for: Qualitative interviews of patients with COPD and muscle weakness enrolled in a clinical trial evaluating a new anabolic treatment: patient perspectives of disease experience, trial participation and outcome assessments
Source: J Patient Rep Outcomes. 2024 Apr 20;8:45. doi: 10.1186/s41687-024-00712-0 (PMC11031513; doi:10.1186/s41687-024-00712-0)
Supplement: Supplementary file 2 — Supplementary Material 2 [file 41687_2024_712_MOESM2_ESM.docx]

**Supplementary Materials**

# **Methods**

## **Stage 1: Concept elicitation interviews**

**Participant inclusion and exclusion criteria**

All participants were required to be 45–80 years of age with clinician-confirmed diagnosis of chronic obstructive pulmonary disease (COPD) (post-bronchodilator forced expiratory volume in 1 second [FEV_1_]/forced vital capacity [FVC] ratio <0.70, and FEV_1_ % predicted of 30–80%) and clinician-confirmed diagnosis of muscle weakness associated with COPD. Participants were also required to be current or former smokers with a ≥10 pack-year smoking history, have a body mass index in the range of 16–32 kg/m^2^ and have sufficient cognitive and linguistic capability for active participation in the interview.

Participants with a history of muscle wasting or weakness or physical limitation secondary to a disease other than COPD, and participants with a history of malignancy that was not in complete remission for ≥2 years (or ≥1 year for non-melanoma skin cancer) were excluded from the study.

**Recruitment and interview procedures**

Participants were recruited via a partner recruitment agency across three sites in the USA (Florida, Illinois [Chicago] and Maryland [Baltimore]). Participants were identified from recruiting clinicians, who obtained informed consent and completed a Case Report Form to confirm eligibility and collect background medical information. The recruitment agency then contacted participants to complete a demographic form and schedule the date and time of the interview.

All interviews were conducted by trained, experienced qualitative interviewers. A semi-structured interview guide was used to explore participants’ conceptualization of muscle weakness, and its impact on physical movements and activities of daily living. All interviews were 30 minutes in duration, audio recorded and transcribed. Each transcript was assessed and participant comments that related directly to the main research questions were highlighted and assigned corresponding concept codes using a coding process.

## **Stage 2: Exit interviews**

### **Trial outcome assessments**

For the 13-week home exercise program participants used the Respercise app. An additional daily physical activity goal was set according to participants’ baseline and daily performance, based on input of daily step counts via a wrist-worn activity tracker (Vivofit; Garmin, Kansas City, USA).

Participants’ physical activity was measured using the daily PROactive Physical Activity in COPD instrument (D-PPAC) [1,2], a hybrid tool comprising a daily questionnaire and measured outputs from a triaxial physical activity monitor (GT9X; Actigraph, GT9X, Pensacola, USA) with the device worn on the waist for 7 days at four time points (screening, baseline [Day ₋9]), Day 56 and Day 80). During these periods, participants also rated their physical activity on a daily basis using the PROactive eDiary [1,2].

Participants also performed physical assessments during site visits, including the incremental shuttle walk test [3] and the endurance shuttle walking test [4-6]. In addition, spirometry was performed according to ATS/ERS guidelines [7]. Inspiratory muscle strength was also assessed by measuring the maximal sniff nasal inspiratory pressure [8]. Lean body mass was assessed using a dual-energy x-ray absorptiometry scan.

## **Stage 2: Exit interviews**

### **Demographic and clinical characteristics**

Mean (standard deviation [SD]) age for participants who participated in the overall trial (n=96) was 65.1 (7.1) years (range: 50–76 years) and similar with the 60-minute interview subgroup (n=32; 64.6 [8.2]; range: 50–76 years), respectively. Mean (SD) age for participants who participated in the 15-minute interview subgroup was slightly higher than the 60-minute subgroup (65.5 [6.5] years). Overall, the proportion of males was lower in the overall trial sample (51.0%) compared with the 60-minute interview subgroup (56.3%). A lower proportion of males participated in the 15-minute interviews (37.1%). Baseline mean (SD) FEV_1_ % predicted for the total overall trial sample was 48.8 (10.8), which was lower than the 60-minute interview subgroup (52.3 [11.3]), but similar to the 15-minute interview group (48.4 [9.8]).

**References**

1. Dobbels F, de Jong C, Drost E, Elberse J, Feridou C, Jacobs L, Rabinovich R, Frei A, Puhan MA, de Boer WI, van der Molen T, Williams K, Pinnock H, Troosters T, Karlsson N, Kulich K, Rüdell K (2014) The PROactive innovative conceptual framework on physical activity. Eur Respir J 44 (5):1223. Doi:10.1183/09031936.00004814

2. Gimeno-Santos E, Raste Y, Demeyer H, Louvaris Z, de Jong C, Rabinovich RA, Hopkinson NS, Polkey MI, Vogiatzis I, Tabberer M, Dobbels F, Ivanoff N, de Boer WI, van der Molen T, Kulich K, Serra I, Basagaña X, Troosters T, Puhan MA, Karlsson N, Garcia-Aymerich J (2015) The PROactive instruments to measure physical activity in patients with chronic obstructive pulmonary disease. Eur Respir J 46 (4):988. Doi:10.1183/09031936.00183014

3. Singh SJ, Morgan MD, Scott S, Walters D, Hardman AE (1992) Development of a shuttle walking test of disability in patients with chronic airways obstruction. Thorax 47 (12):1019-1024. Doi:10.1136/thx.47.12.1019

4. Revill SM, Morgan MD, Singh SJ, Williams J, Hardman AE (1999) The endurance shuttle walk: a new field test for the assessment of endurance capacity in chronic obstructive pulmonary disease. Thorax 54 (3):213-222. Doi:10.1136/thx.54.3.213

5. Holland AE, Spruit MA, Singh SJ (2015) How to carry out a field walking test in chronic respiratory disease. Breathe (Sheff) 11 (2):128-139. Doi:10.1183/20734735.021314

6. Campo LA, Chilingaryan G, Berg K, Paradis B, Mazer B (2006) Validity and reliability of the modified shuttle walk test in patients with chronic obstructive pulmonary disease. Arch Phys Med Rehabil 87:918-922. doi:10.1016/j.apmr.2006.03.005.

7. Miller MR, Hankinson J, Brusasco V, Burgos F, Casaburi R, Coates A, Crapo R, Enright P, van der Grinten CP, Gustafsson P, Jensen R, Johnson DC, MacIntyre N, McKay R, Navajas D, Pedersen OF, Pellegrino R, Viegi G, Wanger J, Force AET (2005) Standardisation of spirometry. Eur Respir J 26 (2):319-338. Doi:10.1183/09031936.05.00034805

8. Uldry C, Janssens JP, de Muralt B, Fitting JW (1997) Sniff nasal inspiratory pressure in patients with chronic obstructive pulmonary disease. Eur Respir J 10 (6):1292. Doi:10.1183/09031936.97.10061292.

**Supplementary Figure 1.** Study schematic.


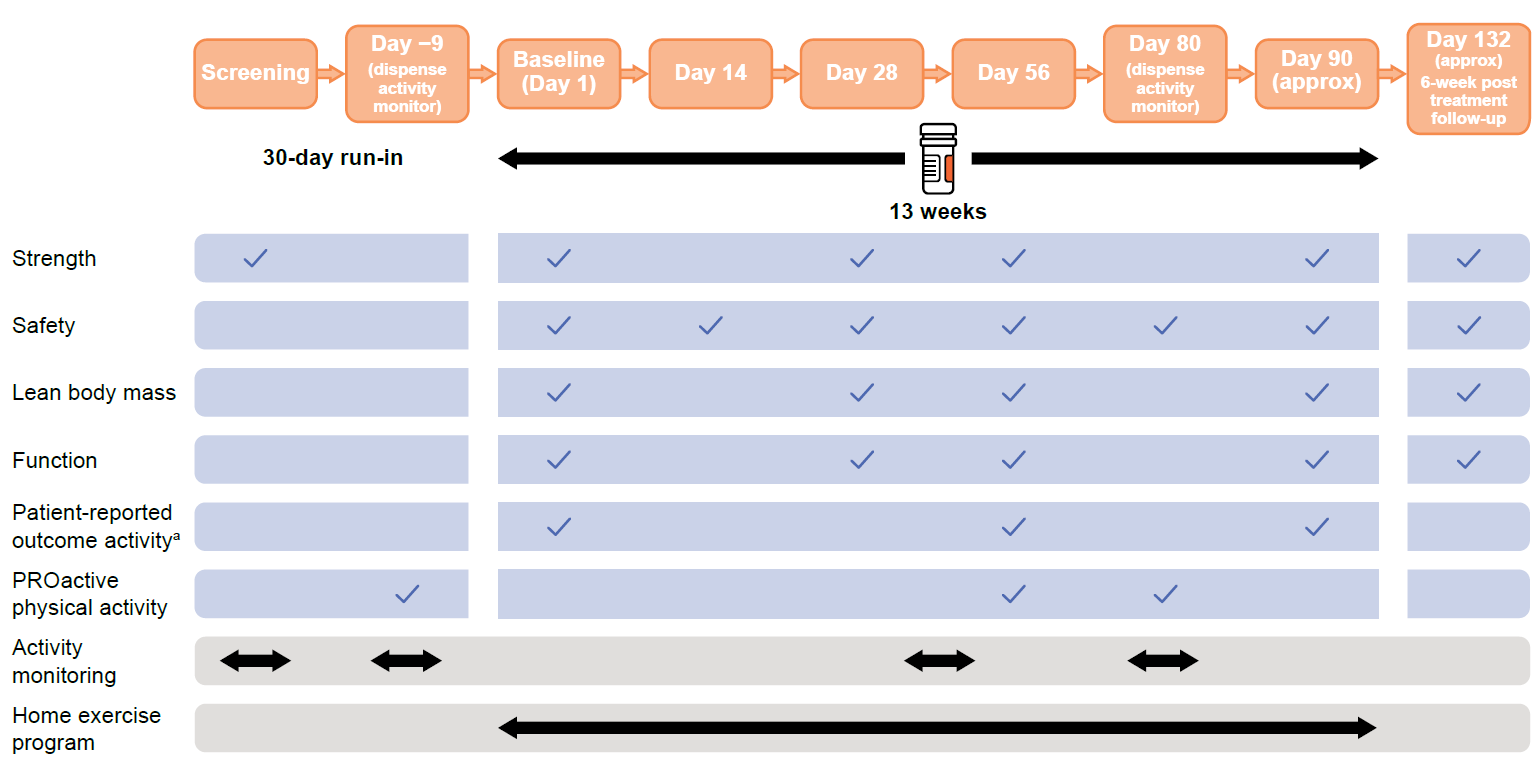
^a^COPD Assessment Test was scheduled on Days 1, 56 and 90; SGRQ was scheduled on Days 1 and 90.

COPD, chronic obstructive pulmonary disease; SGRQ, St George Respiratory Questionnaire-COPD.

## **Supplementary Table 1**. 200182 trial lean muscle body mass, strength, and PRO assessments.

| **Procedure** | **Description** |
| --- | --- |
| DXA | Appendicular, and total lean mass was assessed |
| Handgrip strength | Handgrip strength of both hands was measured and recorded using a hand dynamometer with participants seated, their shoulders adducted, elbows flexed to 90˚ and forearms in a neutral position. Participants were asked to make a maximal effort to squeeze the handle and strength was recorded to the nearest kilogram |
| Leg strength | 1-RM leg press on a pneumatic instrument (A300; Keiser, Fresno, CA, USA) via a standardized procedure |
| SPPB | Participants were assessed for balance, timed rises from a chair, and gait speed, and were scored according to published guidelines |
| ISWT | Maximum distance walked. The greater of the two measurements of distance walked was used to determine walking speed during the ESWT assessment |
| ESWT | Lasted for 20 minutes or more. It could be repeated at the next level (greater walking speed) following a 30-minute rest |
| SGRQ-COPD | A 50-item disease-specific HRQoL questionnaire assessing symptoms, activity limitations and impacts associated with COPD |
| PGRS | A single global question that asked participants to rate their COPD severity on a 4-point scale (mild, moderate, severe, very severe) |
| CAT | To measure overall COPD-related health status for the initial assessment and longitudinal follow-up of patients with COPD |
| PGIC | A single global question that asked participants to rate their general state of health since the start of the study. Responses to the PGIC question were on a 7-point Likert scale ranging from very much improved to very much worse |
| D-PPAC instrument and Physical Activity Monitor | The Daily PROactive instrument is a hybrid outcome assessment tool comprising an electronic diary and outputs from a triaxial physical activity monitor (Actigraph), which has been assessed as compatible with PROactive. Actigraph was used to measure levels of activity and was worn for 7 days by the study participants at 4 time points during the study to provide the total PROactive score |
| Monitored home exercise program | Administered via an application developed by GSK. The application gave customized instruction to participants to incrementally increase their physical activity (via monitoring step counts) and strength (through a combination of strengthening exercises) |

1-RM, one repetition maximum; CAT, COPD Assessment Test; COPD, chronic obstructive pulmonary disease; D-PPAC, Daily PROactive Physical Activity in COPD; DXA, dual-energy X-ray absorptiometry; ESWT, Endurance Shuttle Walk Test; ISWT, Incremental Shuttle Walk Test; PGIC, Patient Global Impression of Change; PGRS, Patient Global Rating of Severity; PRO, patient-reported outcomes; SGRQ, St George Respiratory Questionnaire; SPPB, Short Physical Performance Battery.

## **Supplementary Table 2**. Feedback on the 200182 trial outcomes measures as reported by participants in the 60-minute interviews.

| **Aspect** | **Key findings** | **Supporting quotes** |
| --- | --- | --- |
| **Home exercise program** | | |
| **Likes/dislikes**  **(n=31)^a^** | - 31/31 (100%) participants reported positive aspects of the home exercise program. The program was positively described as: - ‘Good’ (n=11) - ‘Enjoyable’ (n=6) - ‘Helpful’ (n=3) - ‘Fun’ - ‘Exhilarating’ - ‘Enlightening’ - ‘Effective’ - ‘Easy’ - Preferred aspects of the program included: - Resistance band exercises (n=8) - Walking exercises (n=6) - Feeling stronger (n=6) - Increased levels of physical exercise (n=3) - Increased motivation to exercise (n=3) - Increased energy (n=2) - Leg press exercises - Arm exercises - Having an exercise schedule - Flexibility to complete exercises in the participant’s home - Flexibility to complete exercises sitting, standing, or lying down | *“Oh, the exercises were good. I enjoyed the exercise, all of it.”*  *“Home exercise was, was really good. It kind of, uh, enlightened me actually on how important it is to, to, uh, take some time at home and do exercises. So I probably followed the, followed the routine pretty well and, and realized that it actually, it actually makes a difference. Because I have not been one to do, you know, uh, an exercise regimen at home outside of walking and working. So, so I found it to, to be enlightening.”*  **“***All of them just makes you feel better, makes you feel stronger, you know. Got me stronger and I feel like I can feel it, you know, all of it, all the exercises.”*  **INTERVIEWER: *“And what did you like best about those exercises?”*** *“Well, um, the walking because you’re outside, fresh air, um, doing little exercises in the house. Ah, it was good, it was all-round good.”* |
|  | - 7/31 (22.6%) participants described aspects of the home exercise program that they disliked. The least preferred aspects of the home exercise program included: - Walking exercises (n=3) - Stair exercises (n=2) - Standing exercises/rising with resistance bands - Foot press exercises - When the home exercises began - The exercises were ‘boring’ and ‘tedious’ | *“The exercises were simply tedious….I did the exercises, but they were boring.”*  **INTERVIEWER*: “What did you like least about this home exercise program?”*** *“Basically, standing up/rising with the bands because it was the hardest for me. Actually, a reason to do it all the more.”* |
| **Fitting into daily life (n=18)** | - 14/18 (77.8%) participants reported no issues fitting the home exercise program into their daily routine. Examples of when participants completed the program included: - Afterwork (n=2) - After breakfast - 8 am and 8 pm - Before bed - Varied during the trial - No details provided (n=8) | *“Uh, they weren’t hard to do at all and it didn’t take but a few minutes at night before I go to bed.”*  *“I didn’t realize how, how easy it is to do to find the time to exercise.”* |
|  | - 4/18 (22.2%) participants reported difficulties fitting the home exercise program into their daily routine. Reasons provided included: - Difficulty finding time to exercise due to working hours (n=2) - Child minding responsibilities - Lack of daily routine | **INTERVIEWER: *“So, so you managed to get into a routine doing it every day or were there any days where you didn’t complete it?”*** *“Only when, uh—I’m trying to—sometimes I had to work double shifts and I, I didn’t do it on the day I had to do, do a shift at work.”* |
| **Self-reported adherence**  **(n=27)** | - 17/27 (63.0%) participants reported adhering to the home exercise program. Reasons provided included: - Living alone/no distractions - Flexibility in time of completion - No reason provided (n=15) | *“I completed them actually quite dutifully, but not always at the same time. Whenever it was fit best…But I did them.”* |
|  | - 10/27 (37.0%) participants reported not adhering the home exercise program. Reasons for non-adherence included: - Social events distracting participants or reducing their time available to complete exercises (n=3) - Experiencing illness (n=2) - Difficulties fitting the program in around their work schedule (n=2) - Experiencing pain in their legs - No reason provided (n=2). | **INTERVIEWER: *“So was there ever a time where you didn’t complete the exercise program at home?”*** *“Uh, one day I believe, I was ill.”*  *“Well, it was a lot that I had to do that five times a week. But I always did it. But there were days, naturally, when I did not. For example, on my birthday or another birthday, some kind of exceptional thing, I kind of let it slip a bit.”* |
| **Motivation to complete the home exercise program (n=24)** | - 5/24 (20.8%) participants reported that they did not have to ‘push themselves’ to complete the home exercise program. Reasons provided included: - The program was easy - Participant reported that their legs did not get as tired compared with before the trial - A set routine helped participants schedule the program around work - No reason provided (n=2). | **INTERVIEWER: *“And how much did you have to push yourself to do the home exercise program?”*** *“Um, not, not too much. I didn’t have to push myself too much because I just set my mind to doing it when I got home from work every day.”* |
|  | - 19/24 (79.2%) participants reported that they had to ‘push themselves’ to complete the home exercise program. Reasons provided included: - Participants struggled to motivate themselves to start exercising, but this improved with a regular routine (n=8) - Pre-existing health issues limited participants’ capabilities (n=2) - Low motivation (n=2) - Resistance bands were perceived as difficult (n=2) - Stair exercises were perceived as difficult - Feeling tired - Difficulty fitting the program into daily life due to work schedule - Participant reported that it was part of their personality to push themselves to do more than required - No reason provided | **INTERVIEWER: *“So how much did you have to push yourself to do the home exercise program?”*** *“Ah, quite a bit at the beginning. At the beginning it was, it was rather a struggle. But um after a few days, I just got up, did the exercises, I had me breakfast, got dressed, went out and did so many steps, and then continued doing it and increased all the time, so it was very good. I really enjoyed it.”*  **INTERVIEWER: *“Um, the exercise program that you did at home, is, um—can you explain to me how that was?”*** *“Uh, it was good. I had not been doing exercise at home, so I had to kind of, uh, force myself at first, but then I, uh, rather enjoyed doing it.”* |
| **Use of trial device (n=16)^a^** | - 12/16 (75.0%) participants reported no issues using the trial device provided to record their home exercise. Reasons provided included: - The device was easy to use (n=6) - The device was small and portable - No reason provided (n=6) | **INTERVIEWER: *“So the device, you had to use like a mobile device as part of the program. Um, did you find that easy or difficult to use?”*** *“Oh, it was easy to use. It was easy.”* |
|  | - 4/16 (25.0%) participants reported difficulties using the trial device provided to record their home exercise. Reasons provided included: - The device did not work correctly - The device lost internet connectivity - Data was not recorded correctly on the device - The sequence of events on the device was unclear - Experienced difficulty initially when learning how to use the device | **INTERVIEWER: *“Okay. And what about, um, how did you find using the mobile device as part of the home exercise program?”*** *“That was, um, that probably was one of the bad experiences because it, it just never seemed to work properly.”* |
| **Suggested changes**  **(n=19)^a^** | - 14/19 (73.7%) participants did not suggest any changes to the home exercise program | *“Probably wouldn’t change anything.”* |
|  | - 5/19 (26.3%) participants suggested changes to the home exercise program. These included: - Step exercises could be completed by walking up and down stairs - Include more walking exercises outside of participants’ homes - Participant considered the resistance band exercises as necessary but too frequent - Include more exercises to accommodate pre-existing physical restrictions - Increase sets of exercises as participants progress - Provide clearer information of when exercises were to be completed - Include more variety in exercises | **INTERVIEWER: *“Would you change anything if you could?”*** *“Maybe more varied exercises.”*  *“I could not do all the exercises; because of my osteoporosis I could not do all the offered exercises. Well, maybe have a bit more choices, also a bit more varied.”* |
| **Wearable sensors** | | |
| **Usability of wearable sensors (n=21)** | - 21/21 (100%) participants reported that the sensors were easy to use. Positive aspects of the sensors included: - Participants were able to self-monitor their activity (n=5) - Integration into daily life was easy as they were small (n=5) - No issues were experienced - Sensors were easy to use - The wrist monitor felt secure once attached - The wearable sensors were similar to devices participants used previously to monitor walking distance - The wearable sensor was ‘cool’ - No reason provided (n=6) | *“It was informative. I didn’t realize, uh, a few things until I had that walking meter on. Uh, it’s amazing how, how many steps you can take and not realize it. And still be active, you know. It was a good idea.”*  *“It was easy to integrate and to match to the clothing. I was wearing that motion sensor even in the swimming pool. I took it off my belt and fixed it to my panty, that was also no problem.”* |
| **Technical issues (n=31)^a^** | - 22/31 (71.0%) participants reported technical issues and/or negative aspects of the wearable sensors used in the trial. Issues included: - The waist tracker did not feel securely attached to the belt provided (n=11) - Issues saving wearable sensor data in the PROactive daily diary device (n=8) - The waist tracker was uncomfortable, depending on the activity being performed (n=3) - The wrist tracker periodically stopped tracking activity (n=3) - The wrist monitor was perceived as less accurate compared with the waist monitor. Participants reported that when they moved their arm to pick up something or to brush their hair it recorded this as a step (n=2) - Participant sometimes forgot to wear the monitor - The wrist strap broke meaning the participant had to carry the monitor in their pocket - Wearable device malfunctioned. No further detail provided - Wearable devices recorded activity from one appointment to the next rather than daily - No reason provided | **INTERVIEWER: *“So, um, during the study you wore two activity monitors. You had one around your wrist and one on your waist. How did you find using those?”*** *“Yeah. Horrible…the one around me wrist weren't too bad, but the one around me waist, I hate that one…I just struggled to keep it. I was too frightened of losing it more or less, you know, and it became a problem…and it weren't stable enough. So I was feeling it all the time to make sure I still got it.”*  *“I was having problems with that from the beginning and it was kind of confusing for me because it was not zero out. Um, and so I kind of had to write down how many and kind of—I got kind of—knowing when it would zero, like midday. So, um, I don’t feel like I always gave an accurate count.”* |
|  | - 9/31 (29.0%) participants reported no technical issues using the wearable sensors in the trial. No reasons provided. | **INTERVIEWER: *“Were there ever any, um, technical difficulties with either of those?”*** *“No. I didn’t have any.”* |
| **Wrist sensor adherence (n=31)^a^** | - 27/31 (87.1%) participants wore the wrist sensor at all times during the required trial period. No further response was provided. | **INTERVIEWER: *“And did you wear the wrist device on your wrist for the whole treatment period?”*** *“I did.”* |
|  | - 4/31 (12.9%) participants did not wear the wrist sensor at all times during the trial period required. Reasons provided included: - Removing to shower - Left the sensor at the DXA scan site in error   Items worn on the wrist caused the participant to have a skin reaction (pre-trial condition) | *“I wore it—only time I took it off was the, uh—when I took a shower. I didn’t want to get it wet just in case.”*  *“…anything on my wrist for some reason tends to break me out around my wrist and have little bumps under it and they itch. So I, I can't wear anything on my wrist.”* |
| **Waist sensor adherence (n=29)** | - 22/29 (75.9%) participants wore the waist sensor at all times during the required trial period. No further response was provided. | **INTERVIEWER: *“And did you wear the device on your wrist for the full seven days following each visit?”*** *“Yes, ma'am.”* |
|  | - 7/29 (24.1%) participants did not wear the waist sensor at all times during the trial period required. Reasons provided included: - Removing to shower/sleep (n=4) - Lost or misplaced sensor (n=2) - Carried the sensor in their pocket rather than on the belt provided as it wasn’t secure - Participant forgot to put it on some mornings | *“…I did not take it off except to take a shower.”*  *“There was no way to secure it without, uh, without putting it in my pocket.”*  *“There was one time that I lost one…and I had to get another one. But other than that, no.”* |
| **Using two sensors (n=31)** | - 30/31 (96.8%) participants reported not being confused by using two wearable sensors during the trial. Reasons provided included: - The sensors measured two different activities (n=2) - Once wearing the sensors, participants did not need to do anything until recording the readings in the evening (n=2) - The sensors could compare activity levels - Sensors were worn at different locations - No reason provided - 2/30 (6.7%) participants who reported no confusion in using two devices did report not knowing what both sensors recorded, one of whom suggested combining the sensors | **INTERVIEWER: *“Did you find it confusing to have two devices?”*** *“I put them on in the morning and then I simply forgot about it until I recorded the results in the evening.”*  *“No, no, no, no problem at all. One’s on your wrist and the other one’s on your waist so there’s no problem.”* |
|  | - 1/31 (3.2%) participant reported being confused by aspects of the wearable sensors - Participant reported that wearing two sensors was a little confusing at first | **INTERVIEWER: *“And did you ever find it confusing to have two different devices?”*** *“Maybe at first, but then not long into it, it wasn’t confusing at all.”*  *“If you could incorporate both of them as a unit, uh…What's the purpose of the one on the waist?”* |
| **Returning sensors (n=20)** | - 20/20 (100%) participants reported returning the sensors at their next site visit. Reasons provided included: - Returning the sensors was easy as they were worn to site visits (n=2) - No reason provided (n=18) | **INTERVIEWER: *“Did you remember to return the, uh, devices to the site every time?”*** *“Yes, sir. Yeah. Every visit I brought them with me.”* |
| **PROactive daily diary** | | |
| **Usability of daily diary (n=32)^a^** | - 32/32 (100%) participants reported that, overall, the daily diary was easy to complete. Reasons provided included: - The daily diary was ‘easy’; ‘ok’; ‘fine’; and/or ‘no problem’ to complete, and the device was easy to use (n=32) - The daily diary was easy to fit into a daily routine (n=7) - Participants understood the items and the purpose of them within the context of trial (n=3) - The daily diary was ‘cool’ | *“It was fine. It was no problem. I had that, uh, tied to a TV program, so when that program came on TV, it was time for me to, to, uh, fill out that daily diary and it was no problem.”*  ***INTERVIEWER: “So how did you find completing the daily diary questions each day?”*** *“It was very simple. Once I figured out—you know, once you get into the—to a routine again with it, it was very simple.”* |
|  | - 9/32 (28.1%) participants reported negative aspects of the daily diary. These included: - The daily diary was difficult to use at first including not knowing how to respond using the device or how to save/export data to the site (n=6) - Participants were required to adapt to a new routine to complete the diary and/or occasionally found it difficult to find time to complete it (n=6) - Completing the diary and/or hearing the diary alarm were perceived as irritating (n=2) - Daily diary items were repetitive - Participant occasionally forgot to complete the daily diary but was reminded by the alarm - Some items and response options were in a “gray area” (ie, unclear) - The diary was more difficult to complete when traveling or away for work - Participant did not like completing the diary | *“At first, you know, it was kind of like Greek to me, but before long it, it was, it was easy. It wasn’t difficult at all.”*  **INTERVIEWER: *“And so how did you find completing the daily diary questions each day?”*** *“Well like I said, after we got going, um, I mean, you know, got, you know, punch it in the way I was supposed to, uh, there was no problem.”*  *“…it was repetitious and, you know, at some points, you know, in the diary the same questions got the same answers. And—but it was a daily thing and, and, uh, it's something I did to, to comply and just what I do what I'm supposed to do. But yeah, some of the stuff on the diary was, was the same.”*  *“…some of them were kind of a gray area but you just want to make the closest choice as to what answer I wanted to provide.”* |
| **Relevance of items (n=32)^a^** | - 26/32 (81.3%) participants reported that the daily diary items were relevant to their disease experience. Reasons provided included: - Items asked about the participant’s breathing and muscle issues - The items asked were ‘good’ - The items provided a good overview of their disease experience - No reason provided (n=23) | **INTERVIEWER: *“And did you feel like the questions were relevant to your condition?”*** *“It, it was pretty good. It, it—that’s what, you know, where I had my breathing. And the muscle study too.”*  *“I think they were the relevant questions which should be asked to gain a picture of my condition.”* |
|  | - 4/32 (12.5%) participants reported that the daily diary items were partially relevant to their disease experience. Examples included: - Most items were relevant but did not provide further information (n=2) - Items were partially relevant but did not provide further information (n=2) | **INTERVIEWER: *“…did you feel like all the questions in the diary were relevant to your condition?”*** *“Yeah, for the most part, sure.”* |
|  | 2/32 (6.3%) participants reported that the daily diary items were not relevant to their disease experience, stating that they were too focused on activities of daily living instead of disease experience | **INTERVIEWER: *“Did you feel the questions were relevant for you condition?”*** *“No, not really, these were questions on quite everyday things – how much one walks, how demanding it was – so actually no.”* |
| **Suggested changes (n=31)^a^** | - 26/31 (83.9%) participants suggested no changes to the daily diary. Reasons provided included: - Items related to participant’s disease experience and ‘covered’ all aspects (n=3) - No reason provided (n=23) | **INTERVIEWER: *“And would you add anything to the daily diary?”*** *“No. Not right off ‘cause most of it—see it did concern me. I mean it was what I was going through.”*  *“I think you, I think you've got it covered really.”* |
|  | - 11/31 (35.5%) participants suggested changes to the daily diary. Suggestions included: - The item structure/response options did not allow participants to provide a detailed response, suggestion to change to more open-ended responses (n=2) - Increasing the volume of the reminder alarm (n=2) - Change the time when the diary is completed. This included later in the morning and no specific time (n=2) - Change the alarm timing so that it was more frequent or went off later in the evening - Make the introduction to the iPad more concise - Complete every two days instead of daily - Include detailed information about the physical activities participants engaged in - Include items about the weather and/or impacts of weather on their daily experience - Include a measure of heart rate - Include items about trial experience (eg, limitations and expectations) - No details provided | *“I think some, some of them might have been, might have been rephrased and I could give a better answer…so the answer would have been more, uh, clear.”*  *“I think the answer options were not so great. This should maybe be revised. I think it had partially, better, worse and ... yes, constant it had also. Well, I think it should have a more detailed gradation for the answer options.”*  *“…in the summer, when it is very hot, as we had this summer when it was hot continuously over many, many weeks, breathing is harder as when it is not so hot…Now, when it is cooler outside, the breathing is easier right away. And one is able to perform better also. This could maybe be more part of such a study; how the weather was during the study.”*  *“Uh, the alarm on it, you need to make it a little bit louder.”*  *“Uh, probably maybe that we could have just done then when—uh, at random. You know, not had a specific time to do them maybe.”* |
| **Average time taken to complete (n=30)** | - 30/30 (100%) participants reported the average length of time they took to complete the daily diary. These included: - ≤5 minutes (n=21) - 5–10 minutes (n=4) - ‘Not long’ (n=3) - 15 minutes (n=2) | **INTERVIEWER: *“And how long did it take for you to complete the questions each day?*** *“It didn’t even take five minutes.”*  *“Roughly it took, uh, about 15 minutes and then, uh, I had to go back and, and wait an hour after, uh, my—what they call, my alarm went off.”* |
| **Motivation to complete daily diary (n=14)^a^** | - 13/14 (92.9%) participants reported that they did not have to ‘push themselves’ to complete the daily diary. Descriptions provided included: - ‘Not at all’ (n=6) - ‘Not a problem’ (n=2) - ‘Almost none’ - No description provided (n=4) - Reasons provided included: - The diary was not hard to complete (n=2) - The diary was easy to fit into daily life - It became automatic to complete daily - No reason provided (n=9) | **INTERVIEWER: *“Okay. And then how much did you have to push yourself to complete the diary every day?”*** *“Well I didn’t have to push myself at all. It got to be automatic.”*  **INTERVIEWER: *“And how much did you have to push yourself to do the daily diary?”*** *“No, it's no problem.”*  **INTERVIEWER: *“So how much did you have to push yourself to complete that diary?”*** *“Actually none.”* |
|  | - 1/14 (7.1%) participant reported that because they worked long hours they felt that they had to ‘push themselves’ to complete the daily diary. | *“Would have been nicer if one had had two days to record it, because sometimes I was out of the house 10 hours or more.* |
| **Technical issues (4)** | - 4/32 (12.5%) participants reported technical issues relating to the daily diary device. Issues included: - Initial technical issues were experienced with the diary device; however no further detail was provided (n=2) - Short battery life of only 2 hours - Initially the device did not always record all data | *“Well, the diary, the electronic diary, was not working correctly in the beginning as it should have.”*  *“Because the performance of the battery simply was not good; when you did not have it on the power supply, the battery was empty after only two hours.”* |
| **Physical assessments** | | |
| **Likes/dislikes (n=31)^a^** | - 24/31 (77.4%) participants reported positive aspects of the physical assessments completed at site visits. These included: - Physical assessments were perceived as ‘quite good’, ‘good’, ‘okay’, ‘not a problem’ and/or ‘weren’t bad’ (n=12) - Physical assessments were perceived as ‘easy’ (n=6) - Participants liked the walking test (n=5) - It was easy to understand what to do (n=4) - Participants liked the leg press (n=3) - Perceived the physical assessments as fun or ‘a game’ (n=2) - Physical assessments were interesting (n=2) - Physical assessments allowed participants to better understand their physical limits (n=2) - Participant could notice a positive change in his physical abilities - Physical assessments resulted in increased exercise | **INTERVIEWER: *“How did you find completing the physical tests? Were any of them challenging to complete?”*** *“No, completing them was not difficult.”*  *“Um, I enjoyed walking. So, uh, I make it a game, you know, when we have to do a walk from point, point A to point B or to go around the cones. And, you know, I just for my own amusement, I kind of make a game out of it, you know, for my mindset.”*  **INTERVIEWER: *“How did you find the physical tests you were asked to complete during some of the visits?”*** *“Interesting. Well, actually also good for my psyche. To get a feeling for how much you can press or if you are able to walk faster. That was good because I actually do not try this otherwise.”* |
|  | - 22/31 (71.0%) participants reported negative aspects of the physical assessments that were completed at site visits - 16 participants reported that the physical assessments could be difficult and/or challenging to complete. The mostly frequently reported challenging tests were the leg press (n=14) and walking tests (n=5)   - Negative aspects of the leg press included that it was challenging/difficult/hard to complete (n=9); resulted in leg pain (n=2); participants were afraid to press further for fear they could hurt their leg/knee (n=2); participant had issues with breathing   - Negative aspects of the walking test included: the test was not enjoyable (n=2); walking at a faster pace was strenuous/participants were limited in this ability (n=2); breathing issues - Participants found tests tiring (n=3) - Physical assessments in general were not liked (n=2) - The balance/standing tests were not realistic/transferable to daily life - The foot press got ‘really hard’ - The strength tests resulted in the participant being personally disappointed by their lack of strength | *“I wasn’t too crazy about the walking test, but it had to be done.”*  *“Only thing I hated was the leg press... Uh, oh it was hard to do…It was really hard to do.”*  *“I do suffer a little bit with me knees. So I went as far as I thought, yeah that's enough because I don’t want to, you know, do any damage to me knees.”*  *“You know, some of them breathing exercises can get exhausting.”*  *“…the leg strength tests and the strength tests might have been a little disappointing for me…I didn’t have any problem doing the test. It was a little disappointing maybe to me because a lack of strength.”* |
| **Motivation to complete physical assessments (n=23)^a^** | - 14/23 (60.9%) participants reported that they had to push themselves to complete the physical assessments. Descriptions provided included: - ‘A little’; ‘slightly’; ‘not a lot’ (n=7) - ‘Quite a bit’; ‘a lot’; ‘really hard’ (n=5) - Examples of when participants had to push themselves included: - Walking tests (n=4) - All physical assessments (n=3) - Leg press (n=3) - Participants reported that it was part of their personality to push themselves to do more than required (n=3) - During the strength tests | I**NTERVIEWER: *“And how much did you have to push yourself to complete the physical tests?”*** *“Uh, especially for the leg press, I had to push myself pretty hard.”*  *“I pushed myself a little bit on the walking test.”*  **INTERVIEWER: *“Um, great. And how much did you want to push yourself to complete the physical tests, if at all?”*** *“Um, quite a bit. I'm kind of—that's my personality.”* |
|  | - 9/23 (39.1%) participants reported that they did not have to push themselves to complete the physical assessments. Reasons provided included: - The trial site staff encouraged participants to complete tests within their own limits (n=2) - The tests were fun - The tests were easy - No reasons provided (n=5) | *“…the staff made sure that once you reach your threshold they are aware of, you know, what you're doing.”*  **INTERVIEWER: *"How much did you have to push yourself to complete those tests at the visits?”*** *“Oh, none at all. Like I said, it was fun. I wish it wasn’t ending.”* |

^a^Participants provided more than one response so counts exceed totals.

DXA, dual-energy x-ray absorptiometry.

## **Supplementary Table 3.** Summary of symptom experience at the start of the trial as reported by participants in the 60-minute interviews.

| **Aspect** | **Treatment arm responses** | **Placebo arm responses** |
| --- | --- | --- |
| **Muscle weakness** | **Treatment arm (n=9)** | **Placebo arm (n=9)** |
| Description (n=18)^a^ | - 9/9 (100%) participants provided a description of their muscle weakness, using terms such as: - Lack of muscle strength (n=3)   *“…with the exercises that they gave me, it kind of, um, it kind of helped me to, uh, get strength where I had lost strength.”*   - Lack of leg strength (n=3)   *“I haven’t had much leg strength in the past.”*   - Muscle weakness (n=2) - Lack of arm strength (n=2) | - 9/9 (100%) participants provided a description of their muscle weakness, using terms such as: - Lack of leg strength (n=4)   *“I think the exercises and stuff made my legs a little stronger.”*   - Lack of muscle strength (n=3)   *“I knew, um, that my legs were so weak.”*   - Lack of arm strength (n=2) - Muscle numbness in the legs |
| Onset (n=2) | - 2/9 (22.2%) participants described the onset of their muscle weakness occurring a ‘long time’ ago but did not specify how many years they had been experiencing the symptom for.   **INTERVIEWER*: “When did that first start, the muscle weakness?”*** *“Oh, I’ve had that for a long time…probably several years.”* | - |
| Frequency (n=4) | - 3/9 (33.3%) participants described the frequency of their muscle weakness: - Daily (n=2)   *“Every day that I did very much walking and work…then I would get short of breath and, and weak legs and then I'd have to rest.”*   - Once a week   *“Probably once a week or so.”* | - 1/9 (11.1%) participants described experiencing muscle weakness constantly: *“That's all the time.”* |
| Duration (n=3) | - 2/9 (22.2%) participants described the duration of their muscle weakness: - 2–3 days   **INTERVIEWER: *“And when you had it, how long was it lasting for?”*** *“Probably two or three days at a time.”*   - 15–20 minutes   “*Oh, 15–20 minutes.”* | - 1/9 (11.1%) participants described experiencing muscle weakness for ‘a while’ at a time   ***INTERVIEWER: “…you mentioned that your legs ache. Does that stop when you stop walking or does it hurt for a while afterwards?”*** *“Ah, for a while afterwards.”* |
| Bothersome (n=3) | - 2/9 (22.2%) participants described how bothersome their muscle weakness symptom can be: - Sometimes   **INTERVIEWER: *“Did it bother you, your muscle weakness? Did it affect you?”*** *“Well sometimes. Not all the times.”*   - No further information provided | - 1/9 (11.1%) participants described their muscle weakness to be ‘a good bit’ bothersome:   ***INTERVIEWER: “How much does that weakness in your legs bother you?”*** *“A really good bit.”* |
| Impacts due to muscle weakness (n=5)^a^ | - 4/9 (44.4%) participants explained how their muscle weakness impacted on their activities of daily living: - Housework/yard work (n=2)   *“Two or three days at a time. You know, I try to do something at home and I just didn’t have the strength to do it. Yeah.”*   - Shopping   *“I have like a, a—kind of a hill in my backyard and a garden spot and I had to give up my garden spot because I couldn’t walk up and down the hill that good…Out shopping, I couldn’t keep up with other people.*   - Standing from a low chair - Walking uphill | - 1/9 (11.1%) participants explained that they found turning a tap difficult due to their muscle weakness:   *“Before I started…my hot water is hard to turn off in my bath and I usually had to have a washrag…to turn it off.”* |
| **Breathlessness** | **Treatment arm (n=15)** | **Placebo arm (n=12)** |
| Description (n=27) | - 15/15 (100%) participants described breathlessness in a number of ways: - Difficulty breathing (n=5)   *“It’s hard—very hard to breathe, especially some days.”*   - Shortness of breath (n=5)   *“When I walk longer or faster, if you are in a hurry, I just get short of breath.”*   - Out of breath/breathless (n=4) - Lack of air | - 12/12 (100%) participants described breathlessness in a number of ways: - Difficulty breathing (n=4)   *“There are certain things that I just won’t do. I mean it’s, you know, it doesn't make sense to go on a long mountain hike and we live in the mountains, so I don't do that, um, because I know that I’ll have difficulty completing it.”*   - Shortness of breath (n=4)   *“I have to take breaks. Because I am getting short of breath, cannot breathe.”*   - Out of breath/breathless (n=3) - Feel winded |
| Onset (n=10) | - 3/15 (20.0%) participants described when they began experiencing breathlessness: - >10 years ago (n=2)   *“I was diagnosed in 2008 but I, I mean I had been sick like a year before that, didn't know what I had, so it probably started around 2007.”*   - 5–6 years ago | - 7/12 (58.3%) participants described when they began experiencing breathlessness: - 3–4 years ago (n=2)   *“I would say three, four years ago.”*   - >10 years ago   *“I've had it for, for many like years. I'd say at least ten years that I know of.”*   - >20 years ago - A while ago - Prior to diagnosis (time unspecified) - >30 years ago |
| Frequency (n=10) | - 6/15 (40.0%) treated participants described how often they experienced breathlessness. This included: - Depends on workload – with more work increasing frequency of breathlessness (n=2)   *“This all depends on what I was doing. You know, if I had to do a lot of work it was more severe than it was if I was just wasn’t doing nothing.”*   - Several times a week (n=2)   *“Usually two or three times a week I was having some pretty bad breathing spells, you know, and then that lined up to where I wasn’t having them anymore.”*   - 20 times a year - Occasionally | - 4/12 (33.3%) participants described how often they experienced breathlessness, including: - Daily   *“Every day just about.”*   - Several times a week   *“Oh, I feel breathless quite a few times during the week.”*   - 3–4 times a month - Not very often |
| Duration (n=8) | - 3/15 (20.0%) participants explained how long their breathlessness normally lasted for, including: - Up to 10 minutes (n=2)   *“Well maybe five minutes, five or six minutes or something like that.”*   - Doesn’t last long | - 5/12 (41.7%) participants explained how long their breathlessness normally lasted for, including: - Up to 10 minutes (n=2)   **INTERVIEWER: *“How long does it last each time?”*** *“Oh, five or ten minutes.”*   - Until taken inhaler   *“Just until I took my inhaler.”*   - Constant - Lasts a little while |
| Severity (n=7) | - 3/15 (20.0%) participants described the severity of their breathlessness, including: - Bad/very bad (n=2) - *“Sometimes when it start off with it was pretty bad, but right now it’s better now.”* - 3 to 4 out of ten | - 4/12 (33.3%) participants described the severity of their breathlessness, including: - Bad/Severe (n=2)   *“It was, it was bad. It'd get bad. That's why, you know, I ended up going to the doctor about it.”*   - Not ‘really severe’   *“It's not, not really severe where I have to really stop and say, oh I don’t want to do that anymore.”*   - Not severe but difficult to handle   *“Well I would say not real bad, but it was getting difficult.”* |
| Bothersome (n=8) | 1/15 (6.7%) participant described their breathlessness to be bothersome: *“Oh, yeah. It bothered me…”* | - 7/12 (58.3%) participants described how bothersome they found their breathlessness symptom: - Bothersome (n=4)   *“Well it bothered me pretty good 'cause I was still working back when it first started and I'd get out of breath real easy.”*   - Found it scary (000353-F-66-MOD-P)   **INTERVIEWER: *“And how, how bothersome was that breathlessness?”*** *“It kind of scares you a little bit, but I deal with it.”*   - ‘A lot’ - Wasn’t as bothersome as other symptoms but did not specify other symptoms |
| Impacts due to breathlessness (n=17) | - 9/15 (60.0%) reported impacts on their HRQoL due to breathlessness including on: - Walking (n=4)   *“I am short of breath. If I do walk faster than normal or uphill, climb stairs, then I am just short of breath.”*   - Require rests when doing activities (n=3)   *“It don’t last long. You know, I finally get my breathing back. But see like when I do, you know, hard work or something like that I get short of breath*.”   - Nighttime awakenings due to dry mouth, caused by keeping their mouth open to aid breathing - Hospitalization due to breathlessness prior to diagnosis | - 8/12 (66.7%) participants reported impacts on their HRQoL due to breathlessness including on: - Walking (n=3)   *“It means that I cannot walk a thousand steps on the treadmill, I have to take breaks. Because I am getting short of breath, cannot breathe.”*   - Require rests when doing activities (n=3)   *“If I do get really out of breath, then I'll have a minute and then I'll carry on again.”*   - Emotional well-being (n=2) including feeling scared due to breathlessness and depressed due to not being able to do activities the participant wants to. |
| Triggers (n=12) | - 9/15 (60.0%) treated participants explained factors that trigger their breathlessness, including: - Physical activity (n=7)   *“Well, I am short of breath. If I do walk faster than normal or uphill, climb stairs, then I am just short of breath.”*   - Talking with long sentences - Dry mouth when sleeping - Anxiety attacks | - 3/12 (25.0%) participants untreated participants explained that physical activity triggers their breathlessness.   *“…especially if I had done something strenuous or walked a lot or something like that.”* |
| **Fatigue/tiredness** | **Treatment arm (n=7)** | **Placebo arm (n=8)** |
| Description (n=11)^a^ | - 6/7 (85.7%) participants provided descriptions of tiredness/fatigue: - Tired/fatigued (n=3)   *“I was still having a lot of trouble with my breathing, just feeling tired and weak all the time, sluggish, fatigued, but I just got started with everything and I did so much when I had a little bit more energy.”*   - Lack of energy (n=3)   *“I didn’t, I didn’t have a lot of energy, uh, especially in my legs and I have increased energy now, energy levels and my legs feel stronger.”*   - Exhausted - Rundown - Low stamina - Sluggish - Weak | - 5/8 (62.5%) participants provided descriptions of tiredness/fatigue, including: - Tired (n=4)   *“I was tired a whole lot, you know, a whole lot more than what I was when I got done.”*   - Lack of energy - Lethargic |
| Symptom onset  (n=3) | - 2/7 (28.6%) participants described when their fatigue/tiredness began, which included: - Participant could not remember when fatigue/tiredness began but linked the symptom to their breathing   *“I have COPD, so some days I have more energy than others overall and, um, it has to do with my breathing.”*   - One to two years ago   **INTERVIEWER: *“Thinking back to the start of the trial, when did this kind of tiredness and lack of energy first start?”*** *“Oh, gosh. A good while back. Oh, a year or two ago.”* | - 1/8 (12.5%) participant explained that their fatigue/tiredness began **a couple of months ago**   *“I’ve been feeling tired for a couple of months or so.”* |
| Frequency (n=5) | - 2/7 (28.6%) participants described how often they experienced fatigue/tiredness, which included: - Daily   **INTERVIEWER: *“Thinking back to the start, so how often would you experience feeling tired or having no energy?”*** *“Daily. Daily. Every day.”*   - Four times a week | - 3/8 (37.5%) participants described how often they experienced fatigue/tiredness, which included: - Daily   *“Well I felt tired and, and lethargic because I wasn’t doing anything. I had no motivation to do anything. Um, so it was a daily thing. I mean it was a struggle just to get out of bed.”*   - Morning or night - When active |
| Duration (n=3) | - 2/7 (28.6%) participants explained how long their fatigue/tiredness lasted when they experienced it: - For an evening - **INTERVIEWER*: And how long did it [feeling of low energy] last each time?”*** *… “Well there are some nights when I would just, you know, sit in the chair—in my chair and watch TV or something because I didn’t feel like doing anything”* experienced it: - Constant | - 1/8 (12.5%) participant explained that their fatigue/tiredness lasted for a couple of days at a time.   **INTERVIEWER: *“How long would you say that you feel very tired for at a time?”*** *“A couple of days.”* |
| Severity (n=4) | - 3/7 (42.9%) participants described the severity of their fatigue/tiredness: - Moderate   *“It's moderate…It wasn’t severe, most of the time it wasn’t. Just kind of moderate. I mean just a daily tired feeling.”*   - Four out of ten with ten being most severe - ‘Not bad’ | - 1/8 (12.5%) participant untreated participant explained that their fatigue/tiredness was severe as they were tired most of the time   *“Oh, I stay tired. I, I’ve laid around most of the time. I didn’t feel like, you know, getting up and doing anything because I was so tired.”* **INTERVIEWER: *“Okay. So it was quite severe.”*** *“Yeah.”* |
| Bothersome (n=4) | - 3/7 (42.9%) participants explained how bothersome they found their fatigue/tiredness to be: - Fatigue bothers participants ‘a lot’ (n=2)   *“It bothers me a lot. I mean it—you know, everybody wishes they had more energy to get up and go and do…the older I get and more of my symptoms get to bothering me, it, uh, it just brings you down and, uh, you know, I just wish that I had more energy.”*   - Bothers participant as they are not able to do as much as they would like, including activities and attending social events | - 1/8 (12.5%) participant explained that their fatigue/tiredness bothered them a lot:   *“It bothered me a lot because I, um, I watch two children, you know, and me being tired I couldn’t, you know, get up and do things for them.”* |
| Impacts due to fatigue/tiredness (n=8) | - 4/7 (57.1%) treated participants described impacts on their HRQoL due to their fatigue/tiredness: - Participant takes more time to do housework/yard work   *“I had been able to do some yard work and things I wasn’t able to do before. Um, I can do more in the house than I was capable of doing, for longer periods of time.”*   - Requires rest during activities   *“Yes, that is quite exhausting. And not because my arms get tired or so, it is the physical exertion, the breathing, the puffing. I do need to take a bit more time then.”*   - Unable to do activities participant would like and feeling defeated as a result - Lack of motivation to do anything and difficulty falling asleep | - 4/8 (50.0%) participants described impacts on their HRQoL due to their fatigue/tiredness: - Requires rest when participant gets tired   *“…sit down because I get tired..”*   - Difficulty looking after children and being unable to do as much for them as participant would like   *“It bothered me a lot because I, um, I watch two children, you know, and me being tired I couldn’t, you know, get up and do things for them.”*   - Unable to do activities they would like such as attend social events - Lack of motivation to do anything |

^a^Participants provided more than one response so counts exceed totals.
HRQoL, health-related quality of life.
